# Surface Engineering Strategies to Enhance the In Situ Performance of Medical Devices Including Atomic Scale Engineering

**DOI:** 10.3390/ijms222111788

**Published:** 2021-10-30

**Authors:** Afreen Sultana, Mina Zare, Hongrong Luo, Seeram Ramakrishna

**Affiliations:** 1 Center for Nanotechnology & Sustainability, Department of Mechanical Engineering, National University of Singapore, Singapore 117581, Singapore; affo.afreen123@gmail.com (A.S.); seeram@nus.edu.sg (S.R.); 2 Engineering Research Center in Biomaterials, Sichuan University, Chengdu 610064, China

**Keywords:** surface engineering, biomaterials, medical devices, atomic scale engineering, antimicrobial activity, traditional surface engineering, modern surface engineering

## Abstract

Decades of intense scientific research investigations clearly suggest that only a subset of a large number of metals, ceramics, polymers, composites, and nanomaterials are suitable as biomaterials for a growing number of biomedical devices and biomedical uses. However, biomaterials are prone to microbial infection due to *Escherichia coli* (*E. coli*), *Staphylococcus aureus* (*S. aureus*), *Staphylococcus epidermidis* (*S. epidermidis*), hepatitis, tuberculosis, human immunodeficiency virus (HIV), and many more. Hence, a range of surface engineering strategies are devised in order to achieve desired biocompatibility and antimicrobial performance in situ. Surface engineering strategies are a group of techniques that alter or modify the surface properties of the material in order to obtain a product with desired functionalities. There are two categories of surface engineering methods: conventional surface engineering methods (such as coating, bioactive coating, plasma spray coating, hydrothermal, lithography, shot peening, and electrophoretic deposition) and emerging surface engineering methods (laser treatment, robot laser treatment, electrospinning, electrospray, additive manufacturing, and radio frequency magnetron sputtering technique). Atomic-scale engineering, such as chemical vapor deposition, atomic layer etching, plasma immersion ion deposition, and atomic layer deposition, is a subsection of emerging technology that has demonstrated improved control and flexibility at finer length scales than compared to the conventional methods. With the advancements in technologies and the demand for even better control of biomaterial surfaces, research efforts in recent years are aimed at the atomic scale and molecular scale while incorporating functional agents in order to elicit optimal in situ performance. The functional agents include synthetic materials (monolithic ZnO, quaternary ammonium salts, silver nano-clusters, titanium dioxide, and graphene) and natural materials (chitosan, totarol, botanical extracts, and nisin). This review highlights the various strategies of surface engineering of biomaterial including their functional mechanism, applications, and shortcomings. Additionally, this review article emphasizes atomic scale engineering of biomaterials for fabricating antimicrobial biomaterials and explores their challenges.

## 1. Introduction

**Figure 1:**
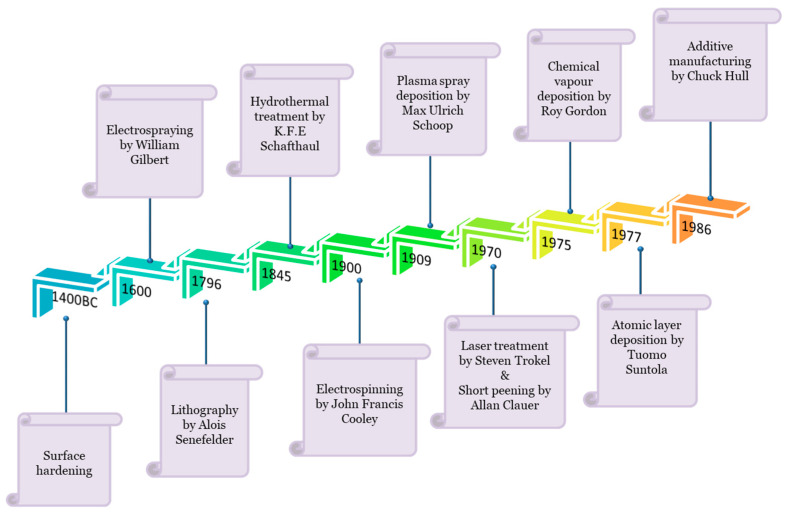
Surface engineering methods have evolved from basic coating techniques to atomic scale deposition methods. This schematic illustration shows the timeline chart of a few surface engineering techniques that are finding biomedical applications.

Surface engineering is a group of techniques used to modify surfaces in two ways: microstructural and compositional modification. Microstructural modification includes shot peening, surface melting, and surface hardening, whereas compositional modification involves coating or deposition (such as physical vapour deposition, plasma spraying, etc.) [1]. Figure 1 represents the timeline chart of diverse surface engineering methods for biomedical devices.

Several mythological tales regarding disabled peoples are reported by Greek, African, Sumerian, and Chinese civilizations. One of the interesting mythologies reported by the Chinese states that Nuwa and Fuxi was a married couple who placed human beings made up of clay in the sun to dry. When the rain came, both hurried to collect the clay structures, but unfortunately some portion of the clay model was lost resulting in the creation of handicapped people. Evidence of implants and prostheses have been found since ancient times; for example, in 2000, Andreas Nerlich found a Cairo toe attached to a right big toe to an Egyptian mummy who lost her natural toe due to diabetes.

**Figure 2:**
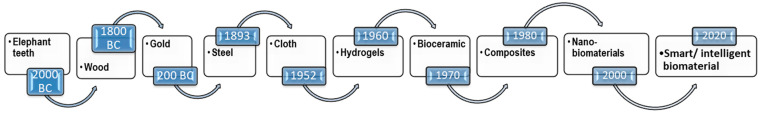
Evolution of biomaterials from ancient prosthetics such as elephant teeth to nano-biomaterials and smart/intelligent biomaterials used in the present era.

First prosthetic eye was developed in 2900 BC. Sometime between 208 and 201 BC, Marcus Sergius had the famous hand prostheses made out of iron, which was capable of holding a shield. In another report, it was mentioned that the Capua leg made up of bronze (around 300 BC) was kept in a college in London, which was destroyed during World War II. Tooth crown made up of bronze was developed in 2nd–4th century AD [2]. In 600 AD, people started using nacre teeth as fashion followed by iron dental implants (around 200 AD). Cautery and sutures (made up of linen, metallic wire, and biting ants) were used for the closure of wounds. An artificial heart was developed by Dr. Paul Winchell during mid-1950s. Most of the implantations studied before 1950 have failed to attain biocompatibility. With the increased focus towards biocompatibility, various biomaterials were researched for intraocular lenses, hip and knee prostheses, artificial kidney and heart, dental and breast implant, vascular grafts, stents, pacemakers, heart valves, and many more [3,4]. Ratner reviewed the history of biomaterials in detail and reported that seventy years ago, there were no materials that existed that were known as biomaterial [3]. Figure 2 represents the evolution of biomaterial from ancient times to the present era.

**Figure 3:**
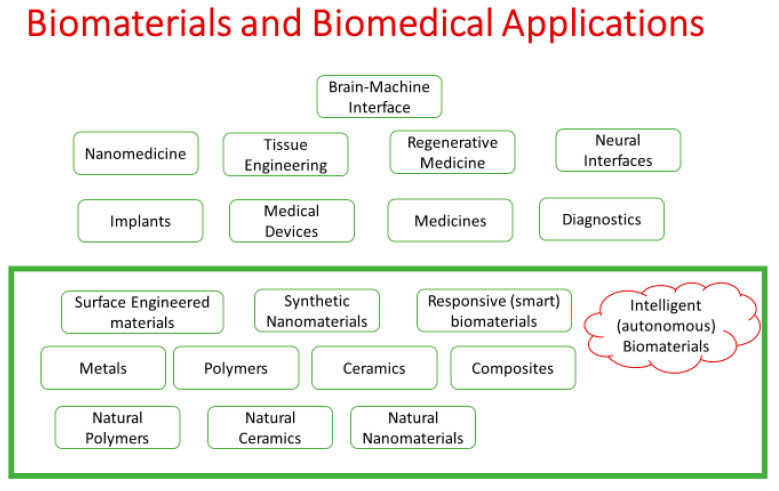
Classification of intelligent (autonomous) biomaterial based on their structure and properties for biomedical applications.

First generation biomaterials were biologically inert [5]. Second generation biomaterials were termed as bioactive biomaterials that formed bonds with tissues, such as glass ceramic [6]. Third generation biomaterials are bioresorbable material that becomes absorbed/degraded by the body itself [5]. Biomaterials are broadly classified into metallic, ceramic, polymeric, composite, and nanobiomaterials [7]. Metallic biomaterials (titanium, cobalt, and steel) provide internal support to the biological tissue and presented applications in orthopaedics, orthodontics, cardiovascular, and neurosurgical devices [8,9]. Biomaterials made up of ceramic consist of two types: bioinert (alumina and zirconia) or bioactive (calcium phosphate and glass-ceramics) [6]. Polymers such as poly(methyl methacrylate), poly(2-hydroxyethyl methacrylate), and polyurethanes are used as biomaterials for the implantation of bones and teeth, soft contact lenses, and heart valves, respectively [10]. The combination of two or more biomaterials results in composite biomaterials such as fiberglass [11]. A special group of biomaterial exists, known as supermolecular biomaterial, which has the beneficial features such as modularity, mechanical tunability, responsiveness, and biomimicry. Applications of supermolecular biomaterial include drug delivery, engineered cell microenvironments, regenerative medicine, and immuno-engineering [12]. The demand for biomaterials that promote the repair, replacement, or restoration of hard and soft tissues continues to grow as the population ages. Intelligent (autonomous) biomaterials can sense a signal, release a specific payload, and adapt their properties to changing conditions in order to keep providing additional, advanced, and/or alternative forms of therapeutics. There are intelligent (autonomous) biomaterials with the innate ability to acquire knowledge (inherent), and they are classified into metals, ceramics/natural ceramics, polymers/natural polymers, composites, natural nanomaterials, surface engineered materials, synthetic nanomaterials, and responsive (smart) biomaterials for biomedical applications such as brain–machine interface, nanomedicine, tissue engineering, regenerative medicine, natural interfaces, implants, medical devices, and diagnosis (Figure 3).

**Figure 4:**
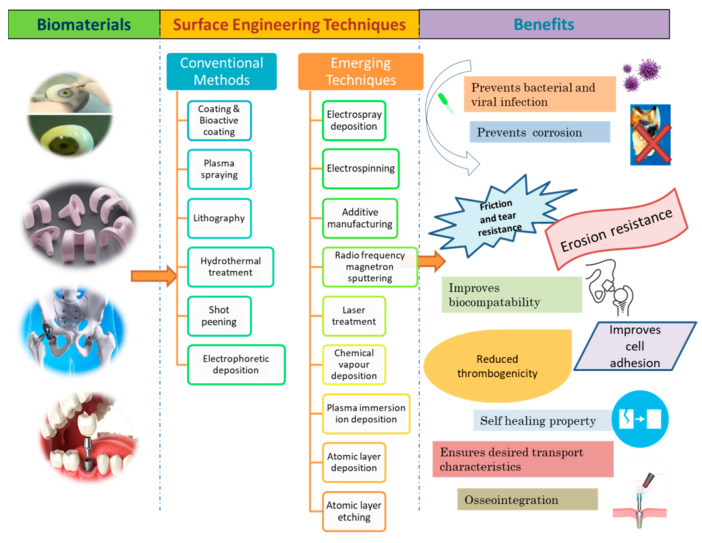
Schematic representation of different kinds of implants along with the few surface engineering techniques that can be used for surface modification in order to attain desired attributes such as antimicrobial, anticorrosive, osseointegration, biocompatibility, and self-healing property.

The significance of biomaterials includes the design and development of scaffolds, neural probes, brain proxies, and brain organoids [13]; the replacement of damaged tissues [14]; and being required in the engineering of the microenvironment to manipulate cancer [15]. Still, there are certain challenges in the utilization of biomaterials such as corrosiveness, brittleness, low stiffness, less uniformity, and infection due to biofilm formation resulting in a second surgery [7,16]. For instance, polymeric biomaterials such as residual methyl methacrylate monomer in poly(methyl methacrylate), silicones, and poly (ethylene terephthalate) in addition to having phenomenal desirable characteristics can cause cell deterioration, eyes and skin infection, disturb blood flow, and induce clots [17,18,19]. In order to counter these shortcomings, surface modifications can be helpful for avoiding undesirable interaction [10]. Enhancement of surface attributes of a material via coating or some other engineering technique is termed as surface engineering [20]. Surface modification has significant role in biomedical application in terms of enhancing osteointegration, preventing corrosion, and inhibiting bacterial infection [21]; to discard ineffectiveness of bactericides due to development of bacterial film [22]; and to create biomaterials with antibacterial and antiviral property [23]. Figure 4 displays the surface modification of biomaterials to prevent microbial contamination and corrosion.

This article includes a short discussion about the steps involved in microbiological deterioration of biomaterial. The aim of this review is to study the surface engineering techniques that can be used to improve antimicrobial activity of biomaterials. Here, we describe the mechanism of engineering techniques and their future prospects. This study also focuses on the atomic scale engineering methods and its challenges. Table 1 displays surface treatment performed on commonly used biomaterials.

## 2. Microbial Interaction with Biomaterial: Biocontaminations in Biomaterials

Biocontamination can be defined as the biological deterioration of material by microbes or due to their toxic by-products [44]. It is necessary to study the prediction of adhesion of microbes to the biomaterial surface because they are the cause of serious hazards. Biocontamination depends on the physiochemical characteristics of microbes and the material of both [45]. Biomaterials are susceptible to biocontaminations that cause infection. Commonly found biocontaminations that deteriorate biomaterial functioning are due to bacteria and virus. Some of them are mentioned in the Table 2.

**Figure 5:**
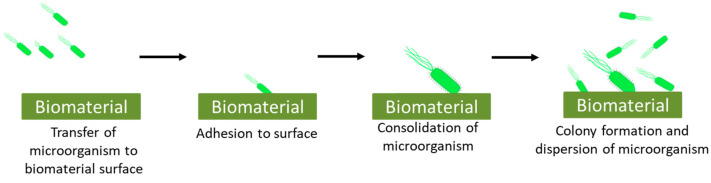
Representation of steps involved in attachment of microorganism to the surface which results in biomaterial related infections.

Biocontamination is defined as the deterioration of material via any biological component that involves a four-step mechanism: transfer of microorganism to the surface, adhesion to the surface, consolidation, and colony formation on the surface (Figure 5) [73].

- Transfer of microorganism:

Transfer of microorganism to implanted devices can occur by either of the following means: hands of members (such as doctors and nurses) performing surgery, unsterilized medical equipment or tools, patient’s own body, patient’s contact with other visitors immediately after the surgery, and remote local infection [74]. Although efforts are made to avoid such inadequacies, a minute negligence could be life threatening.

- Adhesion:

Mechanosensing (potential to mechanically sense physical contact) plays a critical role in the adhesion of microorganisms to the surface [75]. In bacteria, flagellar appendages and pilus acts as a mechanosensors [54,55,56]. Apart from mechanosensing, factors such as surface charge, roughness, hydrophobicity, topography, mechanical stiffness, and chemistry affects the adhesion process [57,58,59,66,67,68,69]. For instance, in a report, the influence of mechanical stiffness on bacterial adhesion was studied using polydimethylsiloxane (PDMS) surface and bacterial strain of *E. coli, S. aureus*, and Pseudomonas aeruginosa (*P. aeruginosa*). For this study, 5:1, 10:1, 20:1, and 40:1 w/w ratio of PDMS to curing agent was considered. The value for Young’s modulus decreased from 4.52 to 0.06 MPa with the increase in PDMS concentration. The contact angle reading suggests that all the samples were hydrophobic, while upon increasing PDMS concentration, the hydrophobicity increased from 109.8 to 120.4. An SEM report showed that all the samples have low roughness. PDMS samples containing bacterial strains were incubated for 2 h at 37 °C in PBS. Moreover, bright field microscopy suggested an increase in number of *E. coli* and *P. aeruginosa* adhesion to PDMS with an increase in softness, while for *S. aureus*, adhesion was significantly the same for all the four concentrations [70]. In another report, the adhesion of *S. aureus* was studied on four surfaces: ultra-high molecular weight poly ethylene (UHMWPE), stainless steel (SS), Ti–6Al–4V alloy, and hydroxyapatite. Among all the surfaces, stainless steel showed the strongest adhesion force while UHMWPE showed the lowest adhesion force. A viability test recorded the values as 65%, 78%, 94%, and 97% for hydroxyapatite, UHMWPE, stainless steel, and Ti–6Al–4V, respectively [71]. UHMWPE is a commonly used biomaterial for arthroplasty, but it usually undergoes microbial infections. The incorporation of vitamin E has the ability to reduce adhesion ability of biocontaminants such as *S. epidermidis*, *S. aureus*, and *E. coli* [72]. Several other studies have reported the role of adhesion on the surface for deteriorating biomaterials [76,77]. After the adhesion of bacteria to the surface, biofilms are formed, which then mature and disperse [78].

- Consolidation:

Furthermore, the microbial cells aggregate on the surface embedded in an extracellular polymeric component and form a cluster. The sophisticated structural characteristics of biofilms help in creating the resistance to surrounding conditions and antimicrobial components. Hence, it can result in chronic and serious biomaterial associated infections. For instance, staphylococcus aureus biofilm formation may result in pneumonia, endocarditis, develops sepsis, skin related infections, and many other biomaterial related infections [79].

- Colony formation:

As favorable conditions such as temperature, humidity, nutrients, and cationic balance are obtained, it results in the formation of a microbial colony due to aggregation and extracellular polymeric substances (consists of components such as proteins, teichoic acid, and lipoteichoic acid). These colonies mature and develop into macro microbial colony, and bacteria become dispersed to a planktonic state [80,81]. It is important to have a better understanding of the interaction of microbes with the biomaterial for designing biomaterial with antimicrobial property that reduces the risk to implant failure.

### Bacterial Infections

Damage of biomaterial quality because of bacterial invasion is a difficult task to treat even with large dosages of antibiotics because it forms biofilms that provide a protective mode for bacteria in hostile environments and protects the bacteria from host defense mechanisms [82]. Bacterial contamination is due to planktonic cells that contribute in the formation of biofilm and sessile cells, which makes it resilient to antibiotics [83]. Once bacteria are transferred to the substrate, it stimulates adhesion by developing signal detecting cells, producing polysaccharides, protein complexes, metabolism, hydrophobicity, viable cells, charge, cell wall stiffness, and appendages [61,62,63,71]. The microstructure of the surface plays a vital role in the adhesion and proliferation of cells and bacteria. A study has suggested improved proliferation of cell and reduction in bacterial adhesion and growth by using submicron-scale manufactured material rather than microscale material [84]. Surface chemistry (especially in the presence of a functional group on the material) has an effect on adhesion kinetics. For instance, in an investigation, self-assembled monolayers of alkanethiols on gold with three functional groups OH, CH_3_, and ethylene glycol were used to study its effect on bacterial (Helicobacter pylori) adhesion, viability, and morphology. It was concluded that the surface with ethylene glycol as a functional group had decreased the viability of adhered Helicobacter pylori [85].

## 3. Host Tissue Reaction: Cellular Responses and Immune Response

### 3.1. Cellular Response of Surface Modified Biomaterial

**Figure 6:**
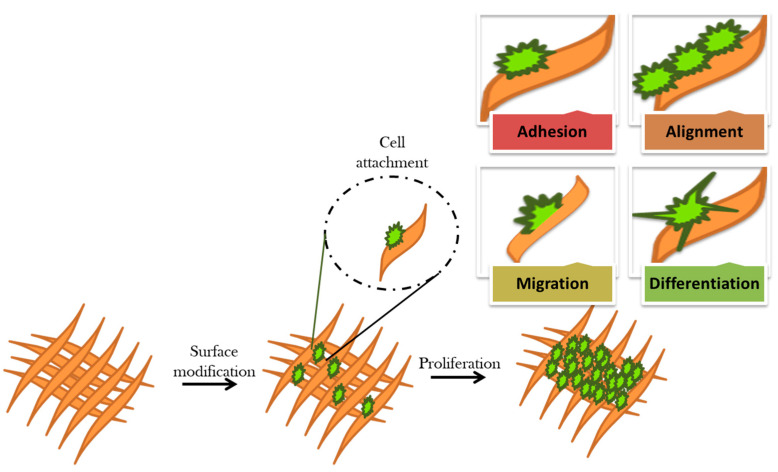
Schematic representation of cellular responses of surface modified biomaterial.

Surface interaction at the biomaterial–cell interface is essential for a variety of cellular functions, such as adhesion, proliferation, and differentiation. Nevertheless, changes in the biointerface enable triggering specific cell signaling and result in different cellular responses. The main aim of the surface modification of biomaterials is to interact with the surrounding tissues and biological fluids and elicit desired cellular responses (Figure 6) [86].

**Figure 7:**
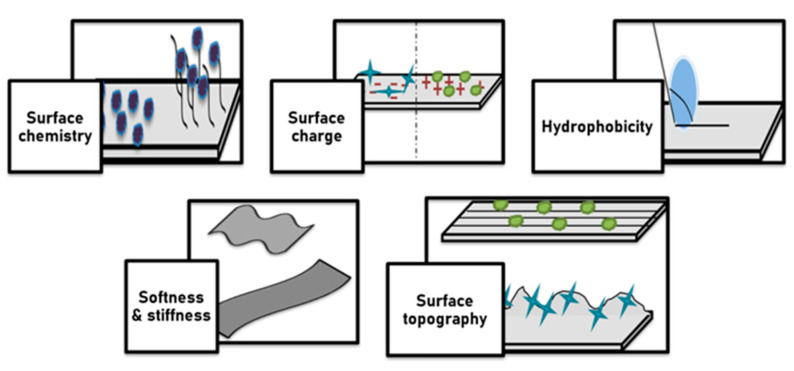
Diagrammatic representation of effect of surface modification of cellular behaviour, which includes changes in surface chemistry, surface charge, hydrophobicity, softness and stiffness, and surface topography (roughness and alignment).

Furthermore, various antimicrobial functionalities are introduced depending on specific applications. For instance, a bone scaffold was prepared by using silver via laser melting technique, which proved to possess the ability for killing 99.9% *S. aureus* within 14 h [87]. Figure 7 displays surface modification and their effect on the cellular behavior.

Based on the types of biomolecule and bioactive agents and properties of biomaterial surfaces that are applied for functionalization, the performance of biomaterials in living tissues changed. Surface modification effects on cellular behavior include surface chemistry, surface charge, hydrophilicity, surface topography, and softness and stiffness of bio-materials. The association of surface chemistry with wettability and surface charge affects cell adhesion, cell shape, cell proliferation, and differentiation. Surface chemistry strongly affects materials’ biocompatibility and immunogenicity. Cells are able to respond to the topographical structure of the underlying surface and modulate their alignment and orientation along the surface. Current nanofabrication and microfabrication techniques for applying surface topography include electron beam lithography or photolithography; replica casting or molding; self-assembling systems; particle synthesis; microcontact printing; sandblasting; electrospinning; and chemical etching. Surface roughness and surface pattern act as the main components of surface topography. Unique properties of surface topography patterns such as high stability, cost-effective manufacture, and easy controllability render them excellent candidates for controlling cell function and tissue regeneration. Solid surfaces can become neutrally, positively, and negatively charged by using different mechanisms. More cells were attached to the positively charged surface compared to the negatively and neutrally charged surfaces. The effects of surface charge on cellular responses depend on the composition of biomaterials, cell type, and tissue microenvironment. Surface wettability (hydrophilicity/hydrophobicity) is the adhesive force between the liquid and solid material surface that causes the spreading of the liquid across a solid surface. It is well documented that proteins tend to bind onto hydrophobic surfaces while cells are typically attached and proliferated on a hydrophilic surface. Surface energy is one of the decisive factors for surface wettability of biomaterials. Surfaces with low surface free energy are less adhesive than those with high surface free energy. Collectively, biomaterials with total surface energies of about 100–129 erg cm^−2^ are more suitable for tissue engineering purposes. Likewise, total surface energies of about 16–20 erg cm^−2^ are within the nonoptimal range to support cell adhesion, proliferation, and differentiation. The stiffnesses of underlying substrate and local extracellular matrix are guiding cell morphology and fate decision.

Implantation for surgical treatments can be performed in three ways: autograft, allograft, and xenograft. Autograft is the process of grafting performed in the same individual; allograft involves implantation in different individuals of the same species; and xenograft refers to grafting between two different species. Among all the grafting techniques, autograft is the most widely used due to lesser chances of graft rejection, but it has limited supply [9]. This shortcoming has shifted the attention towards biomaterials. Biomaterials are materials selected, designed, and processed in order to match with biological fluids or tissues appropriately. Hence, biomaterials have significant importance in the development of medical devices and drug delivery systems [19,88]. For example, they are used for repairing ligaments and tendons, orthopedic applications, cancer therapy, reproduction therapy, nerve regeneration, breast implants, and a range of surgical instrumentation [89].

Earlier, the response of a cell to the implanted biomaterial was considered undesirable; therefore, mostly bio-inert materials were used. However, recent studies focus on materials that have cellular interactions with the biomaterial that encourage adhesion, healing, or cell multiplication.

### 3.2. Immune Response: Natural Instinctive Immune Reaction Following Implantation of Biomaterial

Biomaterial can be regenerative or substitutional. Regenerative biomaterials are those that are capable of restoring the damaged body part. They can be used to fill cavities and for the delivery of curative drugs at the site of spinal cord injury [90]. Substitutional biomaterials are used as a substitute for a body part. However, some of the materials are resorbable and regenerative as well. Then, the interactions with host tissue needs the consideration of degradation by-products, and, for instance, fibrous encapsulation might not results in the best consequence.

After the biomaterial is implanted into the body, injury takes places due to surgical operation which results in the initiation of instinctive immune responses [91]. It includes five stages: adsorption, cell infiltration, cell adhesion, engagement of repair cells, and biomaterial encapsulation. As soon as the biomaterial comes into contact with the tissue, absorption of protein and substitutional material to the biomaterial’s surface takes place [92,93]. This results in coagulation, which is triggered by Factor XII and tissue factor [94]. Factor XII is activated due to the displacement of competing proteins on hydrophilic and hydrophobic surfaces [95,96]. Factor XII releases thrombin, which activates platelets required for coagulation. Moreover, thrombin also breaks fibrinogens to fibrin which creates mesh on the biomaterial surface. Tissue factor initiates the extrinsic system by activating the platelet, resulting in cell infiltration [91].

**Figure 8:**
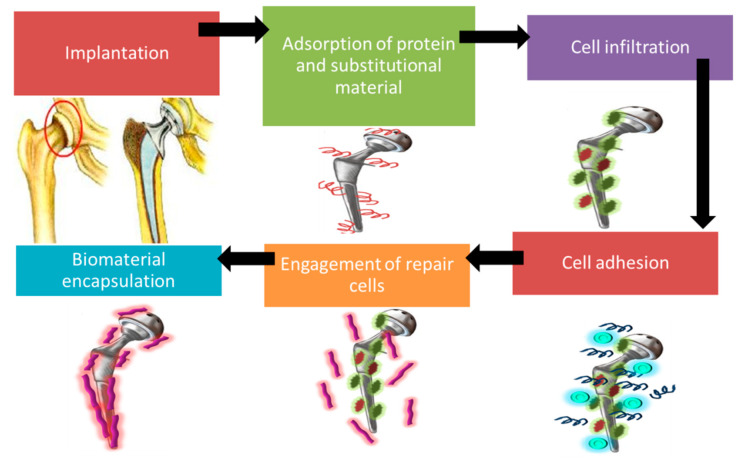
Schematic illustration of natural instinctive immune reaction after a implantation is performed. It includes the adsorption of proteins (occurs at interface level), cell infiltration (occurs only on porous material), cell adhesion which releases cytokines and chemokines, involvement of repair cell, and encapsulation of biomaterial surface.

During the adhesion stage, absorbed proteins that have adhesive receptors promote cell adhesion to the biomaterial surface, which releases cytokines and chemokines. This results in the recruitment of repair cells such as fibroblast. Danger signals such as alarmins are also activated, which triggers immune cell activation [97]. Activated platelets, endothelial cells, and injured cells trigger the release of inflammatory cells (polymorphonuclear leukocytes), which are transferred from blood to the biomaterial surface. Polymorphonuclear leukocytes synthesize immunomodulatory signals such as IL-8, MCP-1, and MIP-1β which activates lymphocytes, monocytes, immature DCs, and macrophages. Within 48 h, polymorphonuclear leukocytes disappear after performing their role [91]. At the last stage, collagen is deposited on the biomaterial surface in order to encapsulate it [98]. Figure 8 represents natural instinctive immune reactions after a implantation is performed.

**Figure 9:**
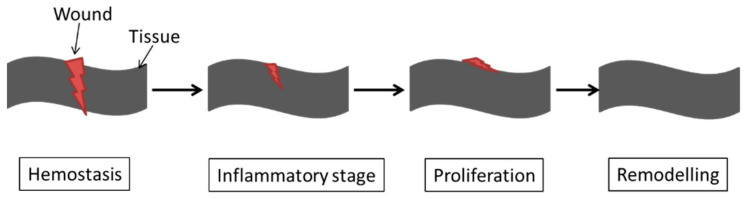
Schematic representation of the four stages involved in wound healing process.

During the wound healing process, four major steps are included: hemostasis, inflammation, proliferation, and restoration. The hemostasis stage involves coagulation, which occurs instantly after the injury along with the inflammatory stage. During the proliferation stage, an extracellular matrix is developed, and remodelling is the restoration that occurs after three weeks of injury [99]. Figure 9 represents the four major steps involved in the wound healing process.

Immune responses are the first responders to foreign biomaterial implantation, which needs to be understood well for designing biomaterial with better biocompatibility, osseointegration, and regeneration.

## 4. Implant Failure: Friction and Wear of Biomaterial Surface

Although implantation has impressive benefits for agonizing patients, it may also result in several complications with time. Loss of appropriate functioning and quality of implant is reported by patients. These implant failures are majorly caused due to friction and wear of biomaterials in addition to biocontamination.

**Figure 10:**
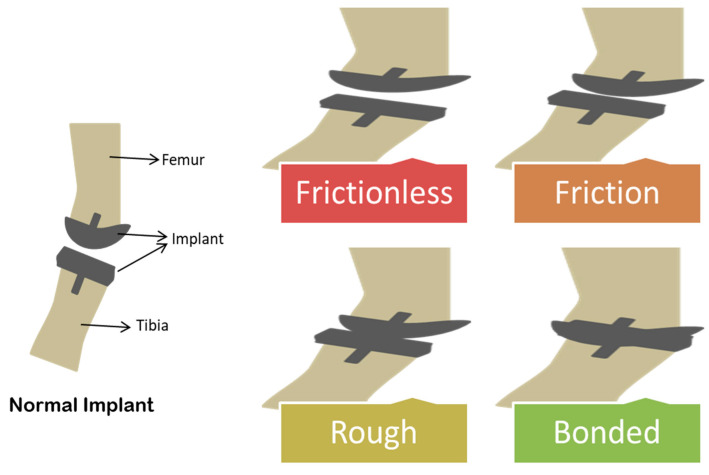
Diagrammatic representation of four kinds of frictional interference in the joint implant: frictionless, friction, rough, and bonded.

After the implant is inserted via surgery, any of the four kinds of interference can occur: frictional, frictionless, rough, or bonded (Figure 10).

Frictionless interference between bone and implant means the free sliding of the implant. Frictional interference is the case where sliding of the implant is limited to a magnitude of the friction coefficient. In rough interference, the implant can slide but cannot be separated, whereas it cannot slide and separate in bonded interference [100]. In a report, it was proved that resting results in friction in hip implants rather than continuous movement [101]. Long term utilization of prosthetic screws causes an increase in friction, which results in implant failure [102]. Friction associated complications can be eliminated by etching and lypolization of the UHMWPE surface. Chitosan consisting of gentamycin was used for this research study with a controlled release system. In coated samples, wear rate and friction were reduced to 19% and 26%, respectively. The release of active component retained for 26 days [103]. A coating of cobalt–chromium condyle was applied on UHMWPE in order to evaluate the effect on the coefficient of friction. The results revealed that the value for the coefficient of friction for coated samples ranged between 0 and 0.15. It was also concluded that a decline in friction is due to an increase in load [104].

**Figure 11:**
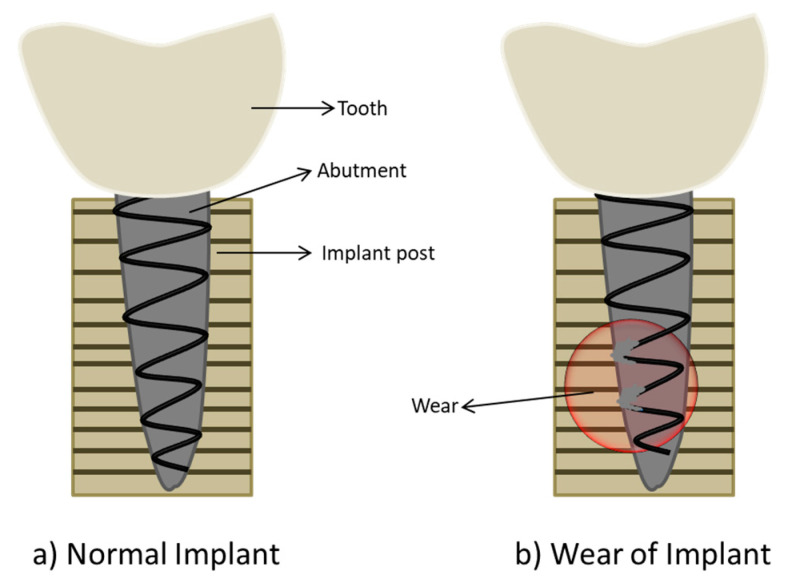
Representation of structural loss of dental implant due to wear. (**a**) represents the normal implant after insertion, and (**b**) represents the wear of implant.

One of the common causes of implant deterioration is reported as mechanical wear [105]. Knee implant failures result in 3.3% of modifying surgeries due to wear and 24.2% due to mechanical loosening [104]. Hip implants may result in osteolysis and the release of metal ions due to wear and corrosion of biomaterials [106]. Figure 11 represents implant failure due to wear of a metallic surface.

Wear related complications in biomedical devices can be cured by treatments such as etching, burnishing, mechanical polishing, laser treatment, milling, and abrasive jet machining [107]. For instance, titanium nitride was coated on Ti-6Al-4V bio-alloy by using direct current reactive magnetron sputtering. A substrate was preheated to 300 °C, and a pressure of 0.38 Pa was considered. Deposition was performed for 175 min, and the thickness obtained was about 5.8 to 6 µm. Two coating were performed, monolayer and gradient coatings, for which the wear rate was recorded as 4.3 × 10^−6^ and 0.6 × 10^−6^ mm^3^/Nm, respectively. Hence, anti-wear property was improved due to coating [108]. In a study, a plasma-assisted chemical vapor deposition was used for coating Cobalt-Chrome-Molybdenum (CoCrMo) with amorphous carbon–hydrogen. Zirconia balls wetted with hyaluronic gel were used to slide against coated and uncoated samples with two frequencies, 50 Hz or 1 Hz. The wear rate was reduced to 0.16 × 10^−6^ mm^3^/N·mat 1 Hz for the coated samples. With the increase in frequency, the wear rate increased to three folds for coated the samples [109]. The incorporation of fatty acid to UHMWPE has the ability to eliminate wear [110].

## 5. Surface Engineering Strategies

**Figure 12:**
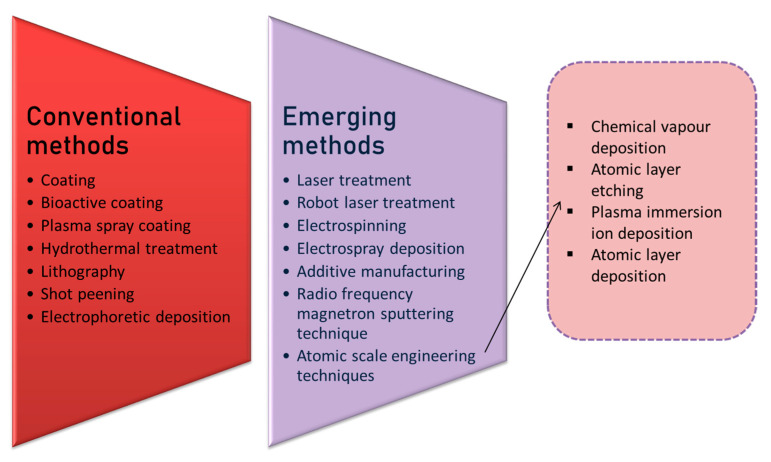
Diagrammatic illustration of commonly used conventional and emerging surface engineering techniques, which include coating, lithography, laser treatment, hydrothermal treatment, plasma spraying, plasma immersion ion deposition, radio frequency magnetron sputtering technique, chemical vapour deposition, atomic layer deposition, electrospray deposition, and electrospinning deposition.

Surface engineering strategies help in rendering the surface tolerant to environmental conditions or external forces that can degrade material quality. The need for surface engineering of material arises when the material undergoes loss of quality due to fatigue (fracture), wear (destruction due to mechanical sliding interaction), corrosion (oxidation of metal surface), or decorative (loss of aesthetic appeal) defects [111]. Surface engineering has the ability to govern cell adhesion, passage, growth, differentiation, and functionality [112]. Surface engineering also has influence on roughness, which plays a critical role in controlling the effectiveness of coating [113]. Some of the conventional and advanced surface engineering strategies applied for modifying biomaterial surface are discussed in this review (Figure 12).

### 5.1. Conventional Surface Engineering Method

#### 5.1.1. Coating

Coating is one of the historic and widely used methods for improving surface properties of a material. The coating of biomaterial with functionalized agents helps to enhance its characteristics [114]. The three common types of coatings performed on biomaterials are polymeric, ceramic, and metallic. The advantages and disadvantages of these coatings are represented in the Table 3.

For instance, in a study, the titanium surface of a dental implant was coated with N-halamine polymer for preventing peri implant infection. The titanium surface was modified in three steps: alkali-heat treatment to develop pore, grafting, and then treating the obtained surface with ethanediamine and sodium hypochlorite. The resulting product is known to be Ti-Poly(acrylic acid)-NCl. Antibacterial analysis for this functionalised surface was studied against *S. aureus* and *Pseudomonas gingivalis*. The results suggest that Ti-PAA-NCl is capable of inhibiting 96% *of S. aureus* and 91% of *P. gingivalis* [120]. In another investigation, silver nanoparticle AgNPs were incorporated in CaP-coated zirconia ceramics. Zirconia ceramics are widely used biomaterials for dental implants but are incapable of binding and interacting with tissue surfaces. For this reason, a calcium phosphate coating is used. Dental implantation causes bacterial colony formation or infection, which has directed the focus to multifunctional coatings of biomaterials. Silver nanoparticles are highly effective against microbial contamination. Therefore, for this study, uniaxial pressing was used to develop zirconia samples followed by incubation in simulated body fluid (comprise of silver nanoparticles) for 3 days in order to obtain a CaP/AgNPs-coatings. Incubation was performed with two methods: by placing the zirconia samples vertically and horizontally in the SBF (stimulated body fluid) with three concentrations of AgNPs (0.1, 0.5, and 3.0 g/L) at 40 °C. Energy Dispersive X-Ray Analysis measurements predicted that Zirconia samples coated horizontally had higher AgNPs deposition. All the samples have shown cytotoxicity except for the samples that were vertically immersed in SBF containing 0.1 g/L AgNPs. The coated samples showed antibacterial activity against *E. coli* and *S. aureus* [121]. Organic coatings such as parylene are also found to have antimicrobial activity. For instance, in an investigation, a titanium disk was dip coated with parylene to examine its inhibitory activity against bacterial adhesion. The cell count assay revealed that parlyene coated samples inhibited the *S. aureus* and *P. aeruginosa* to 3.69 log CFU/mL and 5.51 CFU/mL, respectively. The control samples inhibited *S. aureus* and *P. aeruginosa* to 4.80 log CFU/mL and 6.08 CFU/mL, respectively [24].

Coating can be performed with different methods such as spray coating, dip coating, spin coating, cast-coating, and doctor-blading [122]. The method of coating affects the uniformity and efficacy of the antimicrobial coating. In a study, dip coating and spin coating efficiencies for constructing a chitosan barrier layer on titanium biomaterials were compared. Silk fibroin was mixed with AgNPs and gentamicin to prepare a solution. Furthermore, the experiment was grouped into two subgroups: (i) a group in which a titanium disc of 10 mm  × 10 mm × 0.5 mm dimensions was coated with this solution and (ii) a group in which the disc was coated with the same solution followed by chitosan coating. Chitosan coating was performed with two methods: dip coating and spin coating. From the results, it was observed that both of the coating methods improved bactericidal efficacy to 85.1% and 94.6% for disc coated and spin coated materials, respectively. Comparatively, the spin coated material has better hydrophilicity compared to disc coated material because spin coating showed higher surface smoothness and binding force. Moreover, improved antimicrobial activity was reported in the spin coated samples due to the large number of active protonated amino groups attached to the surface, which brushed off the microbes [22]. Zhou also studied the addition of three different concentrations (6%, 11%, and 18%) of strontium to titanium coating and reported that the highest improvement in the antimicrobial activity against *E. coli* was due to the 11% concentration of strontium [123]. Some of the biomaterials casted with functional substance via simple coating method are mentioned in the Table 4.

##### Bioactive Coating (Incorporation of Antimicrobial Agents to Prevent Biocontamination)

**Figure 13:**
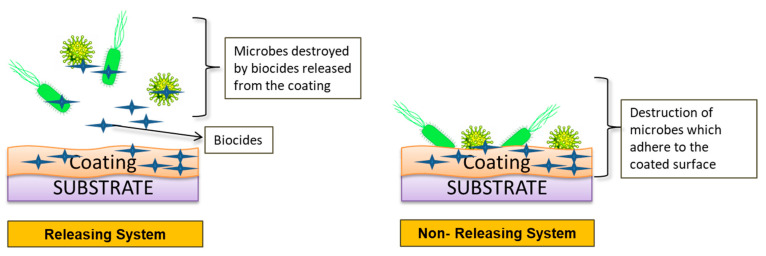
A schematic figure showing the two types of antimicrobial systems: releasing system in which biocides are released into the surroundings of a coated surface to kill microbes and a non-releasing system in which microbes are eradicated when they come into contact with the coated surface.

Surface coating technologies with bioactive agents and biomolecules are commonly used to better mimic tissue microenvironment. Antimicrobial agents are a group of compounds that prohibit the development of microbes [132]. Nanotechnology is an excellent method for incorporating antimicrobial agents in biomaterials by using nanotubes [133], nanowires [134], nanopillars [135], nanospikes [136], and nanoflowers [137]. Microbial contamination of surface can be prevented either via antimicrobial coating (destruction of microorganism when it comes into contact to the surface) or antifouling coating (impedes biofilm formation and/or obstructs microbial accretion). Antimicrobial agents used to prevent biocontaminations are broadly classified into two groups: releasing system and non-releasing system (Figure 13) [138].

- Releasing system:

In this system, coating acts as a carrier of biocides, which become transported to the infected site and inhibits biocontamination and biofilm formation. The releasing system involves the addition of antibiotics, antiseptics, secondary metabolites, nitric oxide, and metals such as silver to the coating. Silver ions and nanoparticles have proven antibiotic effects [139].

- Non-releasing system:

In this system, microorganisms are killed when they come into contact with a coated surface comprising an antimicrobial agent. It includes cationic antimicrobial polymers and photoactive coatings. Cationic antimicrobial polymers agents penetrate into the wall react with microbial membrane, and intracellular matters leach out followed by degradation and cell wall lysis [140]. These agents are further classified into natural (e.g., chitosan) and synthetic (e.g., cationic silicon and poly-acrylates). The effectiveness of chitosan polymer was improved by the incorporation of quaternary ammonium [141]. Polyethylenimine is generally used as a synthetic antimicrobial polymer, which has shown bactericidal activity against various Gram-positive and Gram-negative bacteria [142]. A photoactive coating is composed of materials that become activated in the presence of ultraviolet light and visible light (such as nanoparticles of titanium dioxide and copper oxide). Titanium dioxide co-doped with copper and fluorine has reported the potential to inhibit Methicillin Resistant *S. aureus* (MRSA) [143].

#### 5.1.2. Plasma Spraying Coating

**Figure 14:**
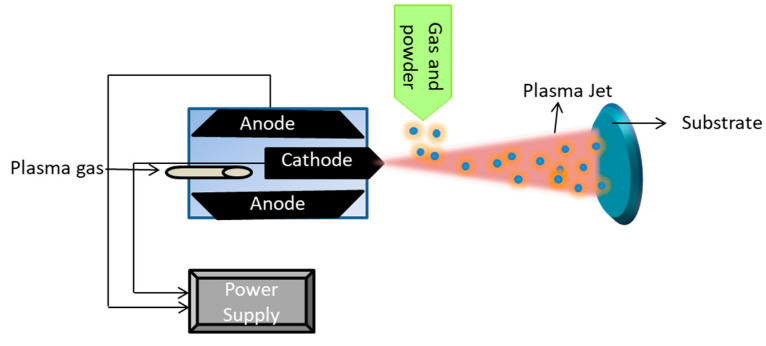
Schematic illustration of plasma spraying process which involves the spraying of powder (material to be coated) in the presence of plasma jet on the substrate. Plasma gas and powdered material are used as inputs.

Plasma spray treatment on biomaterial can be defined as coating of a powder by using a plasma jet at extremely high temperatures (around 10,000 K) on a substrate [144]. This coating can be in molten or semi-molten states [145]. As described in Figure 14, the plasma gas injected towards the surface carries the powdered material, which changes its state from solid to molten or semi-molten during the process and forms a coating on the substrate.

Titanium biomaterials were coated with β-tricalcium phosphate and hydroxyapatite/β-tricalcium phosphate via the plasma spraying method. This method was successful in achieving uniformity and excellent adherence of the coating [146]. In a study, a CoCr (Cobalt Chrome) alloy plate of dimensions 10 mm × 10 mm × 1 mm was coated with silver via the plasma spraying technique. The powder used for coating consists of 3 wt% silver and 97 wt% chrome because a higher percentage of silver can give rise to cytoxicity. An antimicrobial study was conducted against *S. mutants* and *Candida albicans*. In coated samples and a control, the number of colonies of *S. mutants* was recorded at 29 and 198, respectively. The number of colonies of *C. albicans* was observed at 28 and 384 for c the coated samples and control, respectively [147]. A plasma spray coating has several advantages and disadvantages, which are mentioned in the Table 5.

#### 5.1.3. Lithography

**Figure 15:**
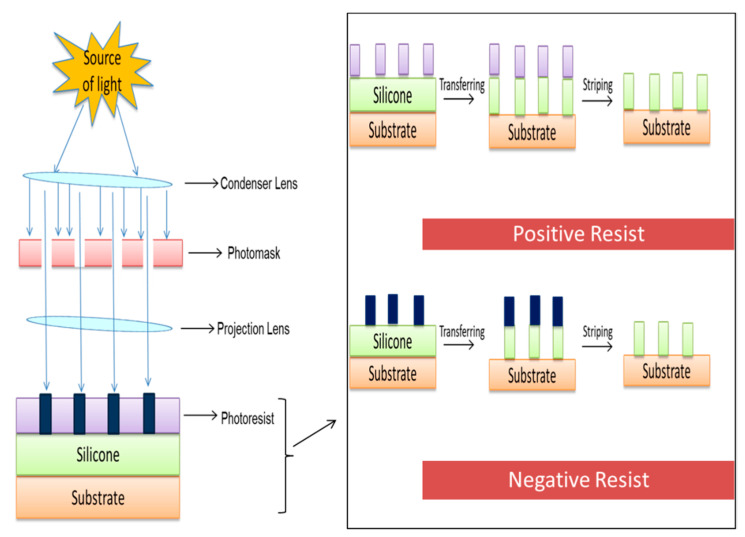
Schematic illustration of surface engineering method demonstrating the basic working principle of lithography on a substrate. Lithography has two results after the rays of source come into contact with a photoresist surface: positive resists and negative resists.

The word ‘lithography’ in Greek is derived from two words: lithos, which means stone, and graphine, which means to write. This technique was introduced by Alois Senefelder in 1976 [150]. Printing using lithography divides the surface into two sections: one is hydrophobic, and other is hydrophilic. The hydrophobic section absorbs the ink while the hydrophilic portion rejects the ink, resulting in a print or pattern [151]. Lithography is a micro and nano fabrication technique of printing on a plane and smooth surface, and it is also known as photolithography [152]. The process of coating a substrate via lithography and its major components is demonstrated in Figure 15.

Photolithography cannot be used for non-planar surface. In order to overcome this issue, a soft lithography method was discovered. Soft lithography is a silicon-based machine which includes an elastomeric mold [153]. In an investigation, titanium dioxide was coated on stainless steel (SS) in order to enhance antimicrobial characteristics. The experiment was divided into three groups: stainless steel polished via soft lithography technique (control); stainless steel coated (SS coated) with titanium dioxide via dipping (5% and 10% concentration); and stainless steel coated (SS micropatterned) with titanium dioxide via dip pen nanolithography (5% and 10% concentration). Surface morphology results showed the contact angle for SS coated with 5% and 10% concentration at 94° and 83°, respectively. For SS coated (5%), SS coated (10%), and SS polished samples, the water angles were recorded at 74°, 74°, and 57°. The roughness of the SS coated surface increased from 180 nm to 197 nm and deceased from 167 nm to 140 nm for the SS microplated sample. The highest adhesion of the Streptococcus mutant was observed in the control (6.1 × 106 CFU/surface), and the lowest was observed in the SS micropatterned sampple (10% TiO_2_ concentration). In the bactericidal study, a 96% reduction in the Streptococcus mutant adhesion to SS micropatterned samples was recorded [154]. The lithograph has several advantages and disadvantages, which are mentioned in the Table 6.

#### 5.1.4. Hydrothermal Treatment

**Figure 16:**
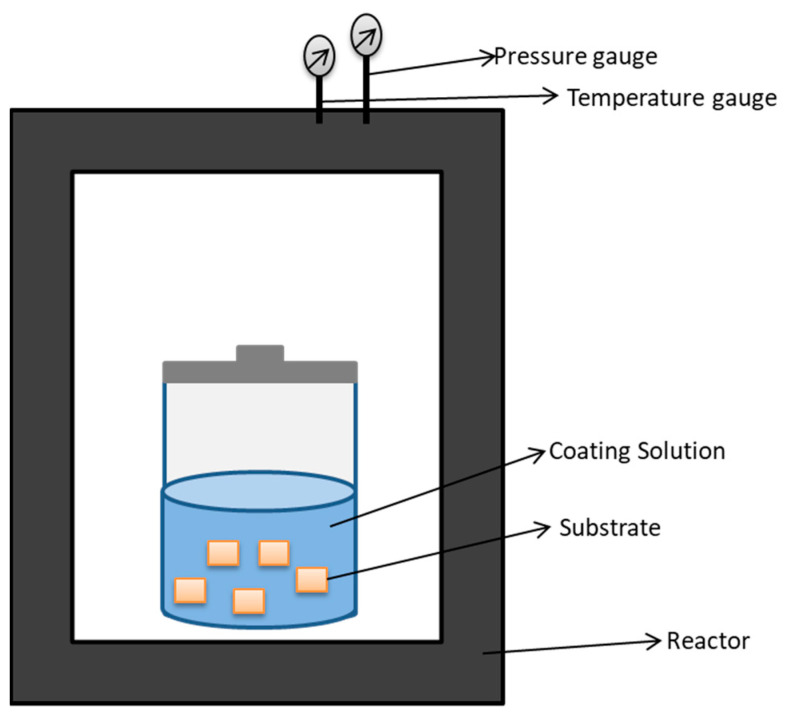
Basic components of hydrothermal treatment include reactor, substrate, coating solution, container with lid to hold the solution, temperature gauge (regulator), and pressure gauge (regulator). Controlled pressure and temperature promote the deposition of film on substrate surface.

Hydrothermal treatment is a process that involves high pressure and high temperature in order to induce a chemical reaction in the presence of water [158]. Figure 16 represents the basic components involved in hydrothermal treatment of substrates.

In a study, a titanium sheet of 12 mm was fabricated with nanostructured TiO_2_ via immersion in 125 mL acid-digestion vessel consisting of 60 mL of 1 M NaOH and was kept at 240 °C for 2 and 3 h, followed by cooling at room temperature, then rinsing with deionized water, and heating at 300 °C for one hour. Furthermore, the sample was treated with 0.6 M HCl for one hour followed by heating at 600 °C (2 h). The experiment was performed in two groups: spear type (2 h of treatment) and pocket type (3 h treatment). Pocket type delayed biofilm formation for up to six days. The results suggest that spear type eliminates bacterial adhesion effectively, and pocket type has shown better antibacterial activity against S. epidermidis [159]. In another study, AgNPs were deposited on the titanium plate (plasma electrolytic oxidised) at 140 °C for 24 h in a vessel via hydrothermal treatment. The treatment was performed in three concentrations of silver acetate solution (0 g/L, 0.01 g/L, or 0.1 g/L). Antimicrobial activities were examined against sessile and planktonic S. epidermidis and *S. aureus*. Antimicrobial activity against sessile S. epidermis was reported at 7.3, 7.3, 4.7, and 2.7 CFU/mL, and the value against sessile *S. aureus* was found at 7.1, 7.2, 4.8, and 2.9 CFU/mL for control, Ag-0, Ag-0.1, and Ag-0.01, respectively. Antimicrobial activity against planktonic S. epidermis was reported at 8.1, 8.0, 5.7, and 4.6 CFU/mL, and the value against sessile *S. aureus* was found at 8.3, 8.2, 5.8, and 4.6 CFU/mL for control, Ag-0, Ag-0.1, and Ag-0.01, respectively [160]. The hydrothermal treatment of biomaterial has several advantages and disadvantages, which are mentioned in the Table 7.

#### 5.1.5. Shot Peening

**Figure 17:**
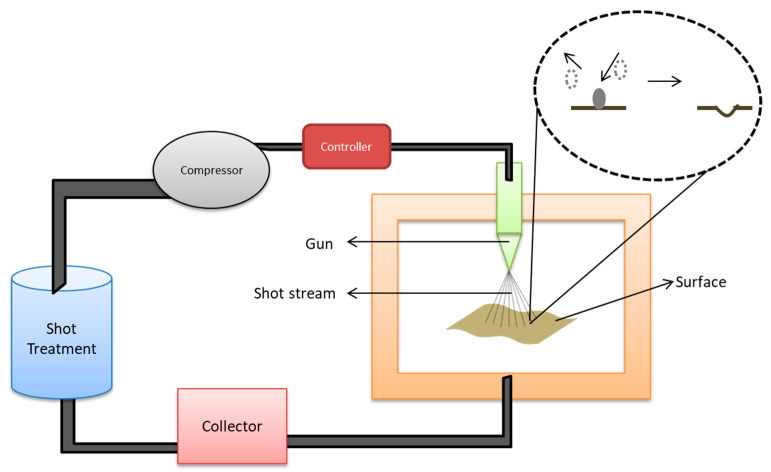
Schematic representation of shot peening process for modifying the surface properties. Compressed air consisting of shot particles is expressed through the gun on the surface which induces a compressive stress. The excess shots are collected and reused.

Shot peening is a process that adds a layer of compressive stress to the surface of material when treated with compressed air consisting of shot particles. The working mechanism includes parts such as a shot container which stores the spherical particles; a compressor to compress air and to accelerate the flow rate of particles; a gun to generate shot stream; and a collector to collect extra shots [164]. Figure 17 represents the schematic illustration of the shot peening process and its major components.

In this process, shot particles strike the surface with force, which causes plastic deformation on the surface. In a study, shot peening has demonstrated the ability to increase resistance to fractures of endodontic files (nickel titanium alloy). This experiment was performed with the distance of 3 mm and an angle of 90° between the nozzle tip and the endodontic surface. Shot peening was performed for 156 s in order to obtain 98% of coverage. The surface roughnesses of treated samples and untreated samples were 0.284 μm and 0.020 μm, respectively. Energy dispersive X-ray spectrophotometric analysis showed that both treated and untreated samples have almost same composition, with the exception that the treated samples possessed a small amount of oxygen (4.57%). Treated samples took 585 s to fracture while untreated samples fractured at 174 s, and the length of fractured fragment was noted as 5.35 and 5.03 for treated and untreated samples, respectively. It was concluded that resistance to fracture was enhanced by hardening of surfaces, plastic deformation, and residues of compression stresses [165]. The shot peening method has several advantages and disadvantages, which are mentioned in the Table 8.

#### 5.1.6. Electrophoretic Deposition

**Figure 18:**
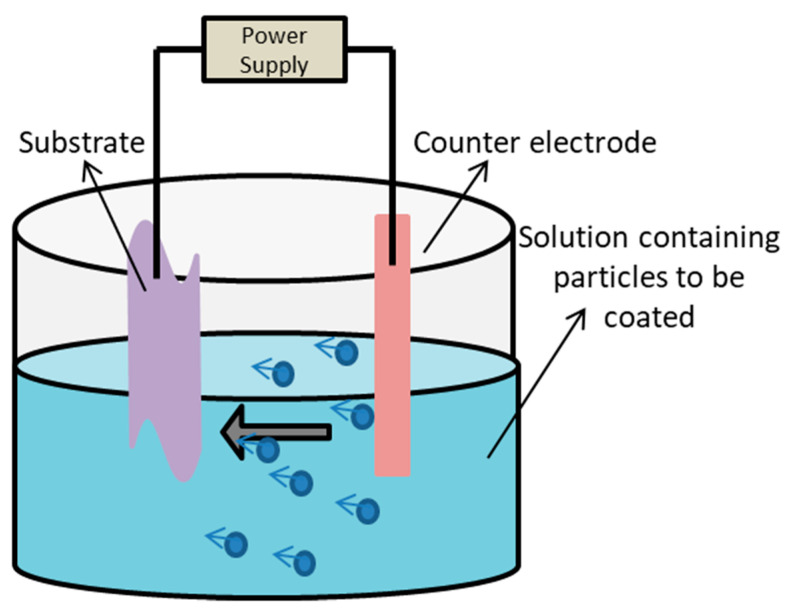
Schematic representation of electrophoresis cell showing movement of coating particles towards the substrate in the presence of electric field.

Electrophoretic deposition is a method that uses an electric field to move colloidal particles suspended in the electrolyte in order to be deposited onto the substrate. Electrophoretic cell consists of anodes, cathodes, electrolytes, and a power supply [168]. Figure 18 represents the diagrammatic illustration of electrophoresis cell.

The biological efficacy of titanium dioxide nanotube fused with type-I collagen was evaluated in a study. The fabrication of titanium dioxide nanotubes on a titanium surface was performed via anodization using copper as a cathode in the presence of ammonium fluoride (0.38 wt%) electrolyte, followed by the incorporation of type-I collagen using electrophoretic fusion in a semi dry transfer system. The experiment was divided into five groups: smooth titanium, nanotube titanium, smooth titanium with chemical linkage to type-I collagen, nanotube titanium with chemical linkage to type-I collagen, and nanotube titanium with electrophoretic fusion. The highest contact angle was observed for nanotube titanium (87.31°), and the lowest was observed for nanotube titanium treated with electrophoretic fusion (23.25°). A platelet aggregation study revealed that nanotube titanium treated with electrophoretic fusion showed the highest platelet-derived growth factor-AB concentration. Moreover, compared to nanotube titanium, the samples treated with electrophoretic fusion had a high number of fibroblasts attached. Hence, it was concluded that electrophoretic fusion of type-I collagen in nanotube titanium can be used to fabricate more robust soft tissue seals. [169]. Electrophoretic deposition has several advantages and disadvantages, which are mentioned in the Table 9.

### 5.2. Emerging Surface Modification Techniques

Emerging surface modification methods are more advanced and innovative techniques for obtaining improved biocompatibility and overcoming challenges faced in conventional modifying techniques. The recent focus is towards machine learning and atomic scale engineering techniques. In machine learning methods, a computer learns the information and data provided and functions according to them [171]. Atomic scale surface engineering is a series of methods that alter the surface topography at the atomic and molecular scale, which is less than 100 nm in size [172]. These techniques help in fabricating a material with improved understanding of surface interactions by modifying internal components [173]. Atomic scale engineering is useful in designing a product with excellent antimicrobial nano-medicine effects [174]. Some of the commonly used atomic scale engineering methods for biomaterial surface modifications are discussed in this review.

#### 5.2.1. Laser Treatment

**Figure 19:**
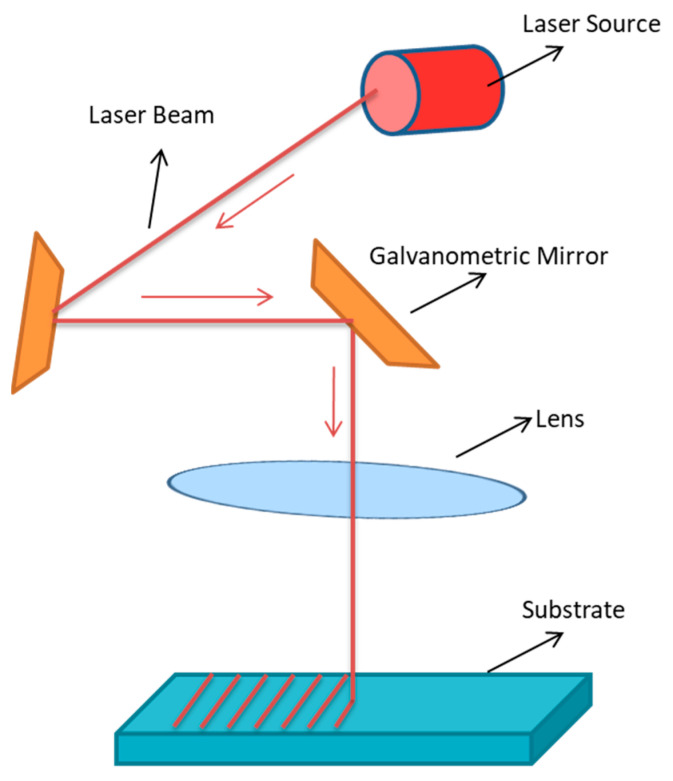
Diagrammatic representation of a substrate undergoing laser treatment and the major components involved in this process. In this process, rays from laser sources hit the galvanometric mirror, followed by lens, and then on the substrate.

Laser treatment is a process that involves using radiation (laser) to modify the surface of a material. The major components and the simple functioning of laser treatments are shown in Figure 19.

Laser treatments are grouped into three types: matrix-assisted pulsed laser evaporation, optical tweezers, and laser capture microdissection [175]. The main advantage of using laser treatment for biomaterial surface modification is the control over thermal penetration and chemical sterilisation [176]. Luo and few others studied the effect of femtosecond laser treatment on a titanium surface with respect to the improvement of bactericidal activity. A titanium surface was treated with 0.49 J/cm^2^ of laser fluence at a speed of 300 mm/s and divided into three groups with 35, 10, and 10 μm intervals for one, one, and two times, respectively. Among all the groups, the control had the lowest contact angle (41.5°), and group two had the highest (58.2°). Titanium surfaces that were laser treated twice showed the highest inhibitory effect (56%) against *E. coli* [177]. In a research study, a stainless steel surface was modified by using laser treatment in three ways: spikes, nano-pillar, and laser-induced periodic surface structures (LIPSS). Treatment conditions were as follows: wavelength of 1030 nm at laser pulses of energy 19.1 μJ (spikes), 1.01 μJ (nano-pillars), and 1.46 μJ (LIPSS) at for 350 fs. Hydrophobicity was measured via contact angle and was found to be 119°, 140°, and 160° for nano-pillar, LIPSS, and spikes, respectively. The antimicrobial study suggests the highest inhibition of *E. coli* and *S. aureus* by LIPSS and lowest by spikes. A reduction in retention of *E. coli* was reported at 99.2% (nano-pillars) and 99.8% (LIPSS). A decrease in retention of *S. aureus* was reported at 79.9% (nano-pillars) and 84.7% (LIPSS). Spikes exhibited worst antimicrobial activity than control (mirror-polished surface). Hence, it was concluded that surface morphology has an impact on the bacterial retention characteristics of a surface [178]. Laser treatments have several advantages and disadvantages, which are mentioned in the Table 10.

#### 5.2.2. Robot Laser Hardening

**Figure 20:**
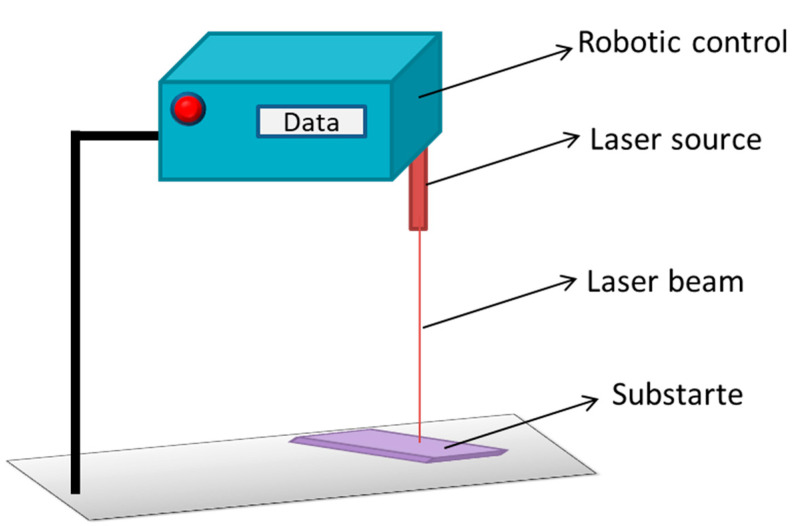
Diagrammatic representation of laser source used to harden the surface of the substrate. The action of laser source is controlled by robot or machine learning.

Laser hardening is a process in which high powered laser beams are used in order to increase surface temperatures above the melting point and then followed by rapid cooling. Robot laser hardening is the addition of machine learning abilities relative to laser hardening techniques by which it is able to perform path related tasks [179]. In a study, a steel surface was treated with the robot laser hardening technique with the temperature range between 850 and 1300 °C and power range between 1000 and 5000 W. The results suggested that a minimum roughness was recorded in the samples treated at 1150 °C and 1000 W, while the maximum roughness was recorded in samples treated at 900 °C and 1500 W [181]. Figure 20 represents the diagrammatic illustration of robot laser hardening technique.

#### 5.2.3. Electrospray

**Figure 21:**
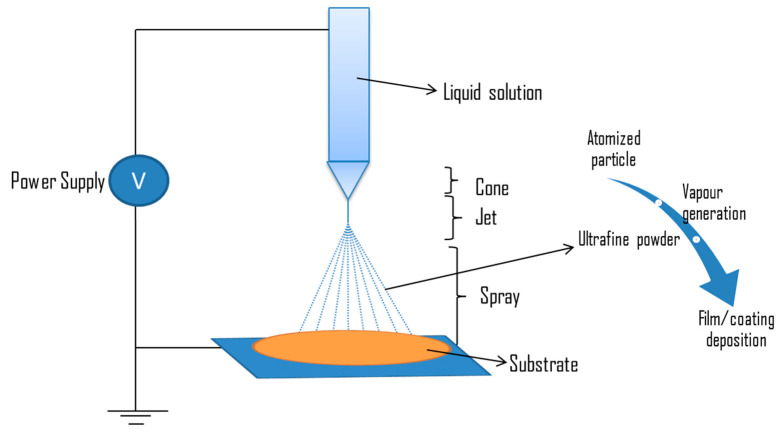
Diagrammatic representation of electrospray deposition of liquid flowing through the nozzle and transformation of liquid solution from atomized particles to vapor, followed by ultrafine powder, and then finally as a film that is deposited on the substrate.

Electrospray can be defined as an advanced technique involving the nanofabrication of functional solution via spraying [182]. It is a process in which high voltages are applied to a liquid flowing through narrow capillaries in order to generate droplets consisting of solutes [183]. During spraying, the liquid is converted to particles, then to vapour, then to ultrafine powder, and finally forms a layer of film [170]. Figure 21 illustrates the electrospray method for coating a substrate.

Factors affecting electrospray deposition include polymer concentration and molecular weight, processing parameters, collection medium, and solvent characteristics [184]. In a research study, a functional solution was incorporated on a titania nanotube substrate via the electrospray deposition technique. The solution was prepared by mixing tetracycline and poly (lactic-co-glycolic acid). This solution was used to coat a titanium disc of 1.2 mm diameter. Electrospray deposition was conducted at a voltage of 25 kV and 10 µL/min of flow rate for seven different time durations (T_0_, T_2_, T_4_, T_8_, T_16_, T_30_, and T_60_) ranging between 0 min and 60 min. The antibacterial report showed that viable cell counts decreased in ESD treated samples, and no microbial growth was found beyond 8 min of treatment. In the control, T_0_, T_2_, and T_4_ colonies, they were reported at 240, 320, 101, and 25 CFU/mL [185]. Table 11 represents some of the biomaterials incorporated with functional agents via the electrospray deposition technique.

Electrospray surface treatments have several advantages and disadvantages, which are mentioned in the Table 12.

#### 5.2.4. Radio Frequency Magnetron Sputtering Technique

**Figure 22:**
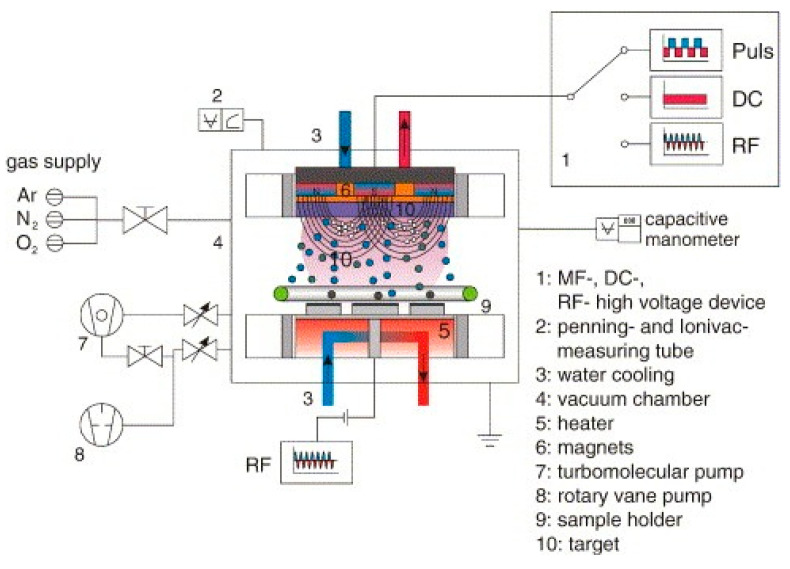
The fundamental parts of radio frequency magnetron sputtering process include radio frequency device, penning, ionivac measuring tube, water cooling, vacuum chamber, heater, magnets, pumps, sample holder, and target. Argon, nitrogen, and oxygen are the three gases used as an input through gas supply inlet [195].

Radio frequency magnetron sputtering method is an advanced technology which works as follows: radio frequency initiates the bombardment of energetic ions on the target surface, then the metal atoms are released from the target into the space, and then followed by deposition of these atoms onto the substrate to form a coating [193,194]. Figure 22 explains the fundamental parts involved in the deposition of substrate via radio frequency magnetron sputtering techniques [195].

Monolithic and hybrid zinc oxide films were treated with Radio Frequency Magnetron Sputtering in order to evaluate the effect on inhibitory activity against *P. aeruginosa* and *S. aureus*. Hybrid ZnO films were prepared by the addition of carbon and copper to it. The research study was categorized into six groups (Zn1, Zn2, Zn3, Zn4, Zn5, and Zn6) with variation including factors such as deposition pressure, time, and power density. Zn5 showed the highest contact angle (96.1°), and Zn6 showed the lowest contact angle (45.5°). Antimicrobial analysis was performed under three conditions: condition A involved in the presence of light; condition B involved in the absence of light; and condition C involved UV treatment in the dark. For *P. aeruginosa*, the results suggest that at condition A, the highest optical density (OD) was found in the control followed by Zn4 (1.01). Moreover, Zn5 and Zn6 recorded the OD as 0.83 and 0.82, respectively. In Zn5 and Zn6 samples, antimicrobial activities were improved due to the presence of carbon and copper. Under condition B, control, Zn4, Zn5, and Zn6 had OD values as 2.14, 0.82, 0.83, and 0.81, respectively. Similar patterns were observed in the inhibition of *S. aureus* under condition A. Under condition B, control and Zn6 OD readings were 2.07 and 0.66. Furthermore, for condition C, Zn4 and Zn6 had the same value (0.70), while Zn5 had (0.66) of OD readings [196]. Radio frequency magnetron sputtering methods have several advantages and disadvantages, which are mentioned in the Table 13.

#### 5.2.5. Atomic Scale Engineering Techniques

##### Chemical Vapour Deposition

**Figure 23:**
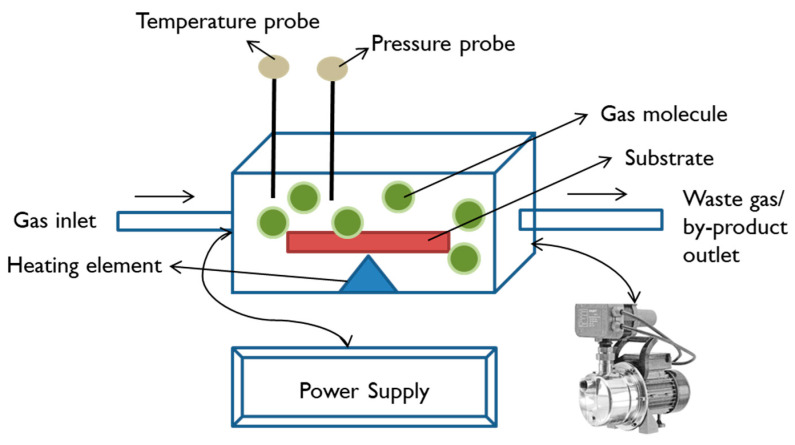
The illustration of coating a substrate via chemical vapour deposition. The components involved in the process include gas inlet, outlet for waste gas, probes for temperature and pressure measurement, heater to increase the substrate temperature, and power supply.

Chemical vapour deposition (CVD) is a coating method in which the vapour phase of solid material is chemically deposited on a heated substrate. In this process, the deposited layer on the substrate is either composed of pure reactant gases or intermediate products formed due to the chemical reactions between gases. The CVD method has several categories based on the advancement in basic features of this process such as pressure (low pressure and atmospheric pressure CVD), method of heating (hot wall and cold wall CVD), direction of gas flow (horizontal and vertical CVD), form of energy used to promote reaction (plasma-enhanced and laser-assisted CVD), and some miscellaneous categories such as metal–organic CVD, hot filament CVD, oxidative CVD, and atomic layer deposition [197]. Figure 23 illustrates the basic working mechanisms of chemical vapour deposition.

Chemical vapour deposition has beneficial properties over other methods, and thick coatings can be easily obtained; high vacuum conditions are not required, and the method is not restricted to line-of-sight coating [198]. This method also has an advantage in that a substrate of any size can be coated [199]. In an investigation, silica coated glass was deposited with ZrO_2_, Cu, and Cu–ZrO_2_ films via chemical vapour deposition methods. Deposition was performed at 430 °C for 45 min at 2 L min^−1^ flow rate in the presence of nitrogen gas. The antimicrobial study showed a reduction in viable cell count to 1.0 log10 and 1.5 log10 CFU for Cu–ZrO_2_ and Cu film against *E. coli* after 15 min of exposure. Viable cell counts reduced to 2 log10 CFU for Cu–ZrO_2_ and Cu film for *S. aureus*. ZrO_2_ films showed no antimicrobial activity [200]. In another research study, polystyrene silicon wafers were functionalised with poly (dimethyl amino methyl styrene), dimethyl amino methyl styrene (DMAMS), and vinyl pyrrolidone (VP) via the chemical vapour deposition method. During the processes, the evaporation temperatures for the functional components were 68 °C (DMAMS), 78 °C (VP), 52 °C (ethylene glycol diacrylate), and 30 °C (tert-butyl peroxide). Temperatures for substrate and filament array were maintained at 40 °C and 240 °C. The experiment was performed in three groups: P(DMAMS-co-EGDA), P(VP-co-EGDA), and P(DE-g-VE) considered as graded coating. The results showed 99.9% inhibition of *E. coli* and *Bacillus subtilis* for the P(DMAMS-co-EGDA) and P(DE-g-VE) functionalized substrates, whereas no inhibition was observed for P(VP-co-EGDA) [201]. Atomic layer etching is a surface modification method that involves the removal of a thin layer from the surface via sequential self-limiting reactions. Modern etching methods include plasma, thermal, and cyro atomic layer etching. In plasma etching methods, chloride ions are absorbed from the surface. Then, the surface is bombarded with activated ions in order to remove the layer. Thermal atomic layer etching includes the formation of surface chemisorption, which is then converted to volatile etching products [202]. In cryo atomic layer etching, the temperature of the sample is reduced below −80 °C and then treated with octafluorocyclobutane followed by bombardment with plasma to create etching [203]. Some of the functional agents deposited on biomaterials via chemical deposition method are mentioned in the Table 14.

Chemical vapour deposition has several advantages and disadvantages, which are mentioned in the Table 15.

##### Atomic Layer Deposition

**Figure 24:**
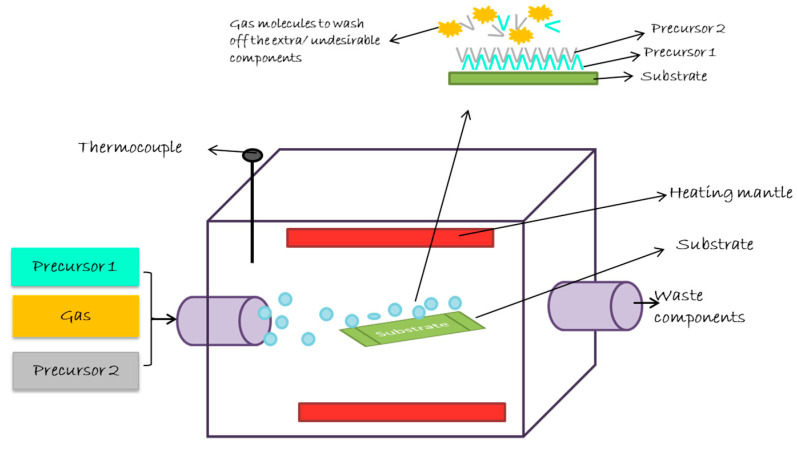
Schematic illustration of working mechanism of atomic layer deposition to coat a substrate. The major components of this process are as follows: two precursors, gas, thermocouple, heating mantle, and inlet and outlet for gaseous compounds.

Atomic layer deposition is a method for pinhole free gaseous thin film deposition at low temperatures [216]. In this method, two precursor and carrier gas acts as an initiator for the reaction. At first, the substrate is treated with precursor 1 and then the carrier gas is used to purge the excess precursor and by-products. Then, precursor 2 is pulsed to treat the substrate, followed by passing the carrier gas to remove excess precursor and by-products. This is repeated until the desired thickness of film on the substrate is achieved [217]. Figure 24 is used to explain the functioning of atomic layer deposition techniques.

Atomic layer deposition is classified into two categories: surface activation and surface blocking. Surface activation enhances the density of active sites and defects which has influence on nucleation and can be performed directly or indirectly. Surface blocking has the ability to selectively block the sites by weakening dissociative chemisorption of co-reactant molecules [218]. In an investigation, zinc oxide was coated on nano-porous alumina membranes via the atomic layer deposition technique. During this process, the temperature was set to 20 °C, and the pressure was at ~0.2 Torr for 6 s duration. Total numbers of deposition cycle were considered at 31. The results suggest that *S. aureus* was inhibited 10 times more on the coated surface than on the uncoated surface. For *E. coli*, uncoated sample inhibited three times lesser than coated samples [219]. Liu, Bhatia, and Webster investigated the atomic layer deposition of titanium dioxide on titanium (1 × 1 cm^2^) substrate at three different temperature conditions (120 °C, 160 °C, and 190 °C) and a flow rate of 100 sccm (standard cubic centimetre per minute). This process included a total number of 2500 deposition cycles. At 160 °C, the coated sample inhibited more than 80% growth of *E.coli*, *S. aureus*, and methicillin-resistant *Staphylococcus aureus*. At 190 °C, the coated sample more efficiently inhibited *E. coli* and poor inhibition of *S. aureus* was observed. At 120 °C, the coated samples showed highest inhibition towards *S. aureus* and least for MRSA [220]. Atomic layer deposition of biomaterial has several advantages and disadvantages, which are mentioned in the Table 16.

##### Plasma Immersion Ion Deposition

**Figure 25:**
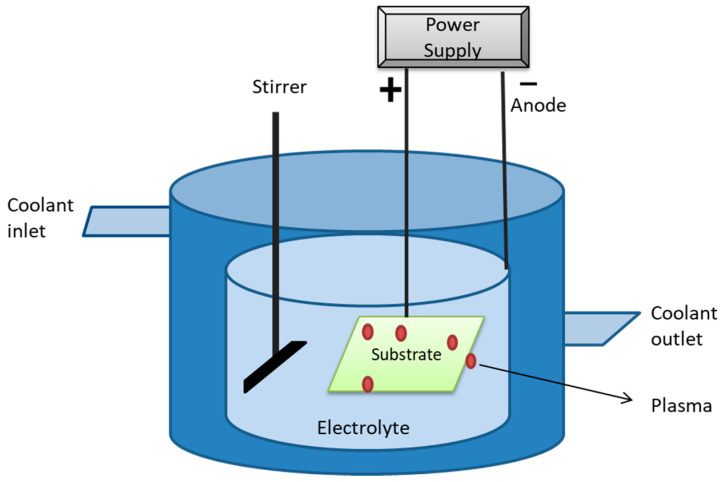
Schematic illustration of working principle of plasma immersion ion deposition technique. It includes a coolant inlet and outlet to maintain flow, stirrer to maintain uniformity, substrate dipped in electrolyte containing plasma, and power supply.

As the name suggests, in this method, the ions of a target are deposited onto the substrate (which acts as a cathode) while being immersed in plasma under low pressure conditions [225]. The functioning of plasma immersion ion deposition is explained with the help of the diagram in Figure 25.

In this deposition technique, the substrate is placed in the vacuum chamber, which produces plasma and comprises the component to be coated. Then, the substrate is treated with high negative voltage in order to deposit the plasma, which results in repelling electrons towards the wall of chamber (at ground potential). A voltage difference is created, which accelerates the ions and promotes embedment on the substrate’s surface [226]. In a study, polyethylene (PE) was coated with Ag and copper (Cu) via the plasma immersion ion deposition method. The results showed that *S. epidermis* was reduced to 2.2 log level for Ag samples whereas no significant differences were reported for Cu samples. It was concluded that Ag deposited PE via plasma immersion ion deposition method is an efficient technique to prevent implant infections [227]. In another study, a titanium disk of 16 mm diameter and 1 mm thickness was treated via the plasma immersion ion method in order to enhance antibacterial properties of the titanium biomaterial. The experiment was conducted in three groups: TC was considered as untreated titanium disk, TL was considered as titanium disk treated with low oxygen ion dose (1 × 1016) for 12 min, and TH was marked as titanium disk treated with high oxygen ion dose (4 × 1016) for 40 min. Other factors such as voltage (30 kV), pulse duration (20 μs), and frequency (200 Hz) were the same for both groups TH and TL. The contact angle value was recorded as 54, 51, and 52 for TC, TL, and TH. Resistance to bacterial adhesion was evaluated against Streptococcus mutants (usually contaminates the teeth surface). After 6 h of incubation, it was observed that 70% of lesser bacteria adhered to the coated surface. Colonies found on the TH samples were lesser than 0.5 × 10^6^ CFU/mL, and for control, the values were higher than 2.5 × 10^6^ CFU/mL [228]. Plasma immersion ion deposition of biomaterial has several advantages and disadvantages, which are mentioned in the Table 17.

## 6. Future Scope of Surface Engineered Biomaterials

**Figure 26:**
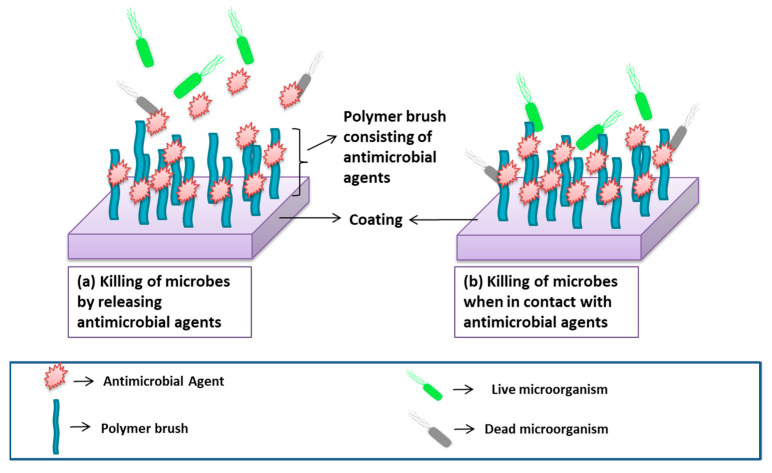
The schematic demonstration of coating consisting of two types of polymer brushes: (**a**) releases antimicrobial agents to kill the microbes and (**b**) kills microbes when in contact with surface.

The incorporation of substance with antimicrobial agents relative to a biomaterial is achievable via surface coating or modifying techniques with advanced strategies such as aniodization [231], thermal spraying [232], atmospheric pressure chemical vapour deposition [233], gel–vapour deposition [234], atomic layer deposition [235], and plasma ionization [236]. Surface engineering techniques help in the fabrication of lightweight and durable fibers. The thicknesses of coating can easily be controlled, and coating of complex structures can be performed. However, due to several challenges such as high temperature, slow deposition rate, and the production of toxic by-products, some techniques can be used for 2D coating, which limits the use of these techniques for commercial applications. Recent studies have reported the innovative approach for improving antimicrobial activities of biomaterial known as passive coating. Passive coatings function by decreasing non-specific interactions and cell attachment to the biomaterial surface [237]. The incorporation of polymer brushes (passive coating) consisting of antimicrobial agents to the surface is one of the advanced methods for preventing microbial adhesion [21,238,239]. Figure 26 illustrates two methods for the functioning of passive coating consisting of antimicrobial agents.

**Figure 27:**
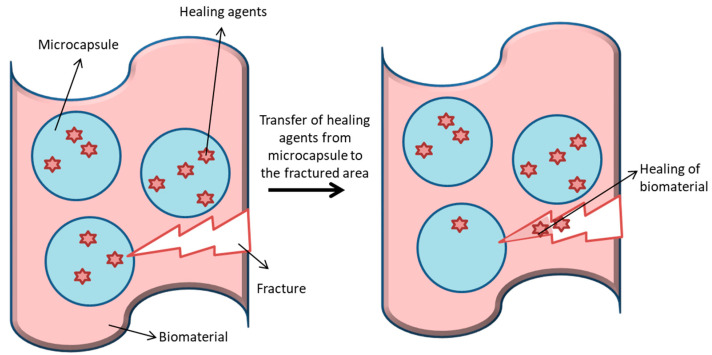
Representation of self-healing property fabricated by using surface engineering technology. The figure shows the fracture of biomaterial surface and the transfer of healing agents from the microcapsule to the fractured area for fast recovery without the requirement of any external stimuli.

Similarly, stimuli-responsive materials are invented, and these materials respond to changes in chemical (such as pH), biological (such as enzymes), and physical (such as temperature) stimuli in a manner in order to combat diseases [240]. These stimuli act as a trigger to provoke a response or change. In some reports, stimuli-responsive material and smart and intelligent biomaterials are considered as identical, and some consider smart or stimuli responsive material and intelligent biomaterial differently. A better method to define smart biomaterial is via four classes of smart biomaterial, which include inert, active, responsive, and autonomous. The inert group consists of material that are harmless. The active group has capabilities in releasing therapeutics in an unregulated manner. The responsive group has the ability to sense the signals and react to them. The autonomous group senses the signals, then responds to them, and then adapts to the altering environment [241]. In general, intelligent biomaterials refer to those that possess instructive or inductive effects on cells and tissues by engineering the material’s responsiveness towards an internal or external stimulus. Intelligent biomaterials have the ability to respond to the changes in physiological parameters and exogenous stimuli and continue to impact many aspects of modern medicine, such as self-healing biomaterials that are capable of regaining their original properties after damage, specifically in terms of mechanical properties, and can help in the fast recovery of damaged tissues [242,243]. Self-healing biomaterials function in two ways: extrinsic and intrinsic. Extrinsic self-healing involves the incorporation of healing agents embedded in a capsule in the material, and intrinsic self-healing does not involve any addition of external healing agents [244,245]. Figure 27 represents the self-healing process of biomaterial when it undergoes fractures.

## 7. Conclusions

Biomaterials with magnificent importance in the biomedical field are still facing challenges due to contaminations resulting in life threatening impact on humans. The commonly used biomaterials include stainless steel, titanium, polystyrene, and bio-ceramic materials. With the increase in demand for natural biomaterials, interests are shifted towards materials such as chitosan, cellulose acetate, and gelatin. The risk of contamination is higher in natural biomaterials due to the presence of nutrients desired for microbial growth and bio film formation. Infection in biomaterials may be a result of improper and unsterilized pre-surgery handling, mishandling during surgery, and post-surgery negligence. Biomaterial-associated infection is an intricate issue because it becomes difficult to treat when using antibiotics, and the only solution remaining is to perform another surgery, which is costly and can cause increased distress to the patient.

Efforts are made to enhance the surface characteristics of the biomaterial in order to inhibit microbial growth either by releasing antimicrobial components or by preventing the adhesion of microbes to the functionalised surface. The role of surface engineering is of great importance in this area, which is defined as any technique involved in the modification of surface properties of materials in order to obtain the desired attributes. Surface engineering plays a vital role in preserving the attributes of biomaterial and enhancing its effectiveness and sustainability by adding a layer (or component) to the surface or changing the surface texture (such as in the case of shot peeing). It helps in preventing corrosion and erosion, promotes osseointegration, enhances biocompatibility, has self-healing property, resists friction and wear, improves cell adhesion, decreases thrombogenicity, ensures desired transport characteristics, and prevents the risk of microbial infections. This review provides all the necessary information about various conventional and emerging surface engineering methods including atomic scale engineering resulting in the enhancement of antimicrobial activity. Innovation in surface engineering techniques can help attain promising biomaterials with strong and long-lasting quality for combating present challenges.

## Figures and Tables

**Table 1:** Surface treatment of widely used biomaterials to obtain desired functional characteristics.

Biomaterials	Surface Treatments	Results	References
Pacemaker	Parylene coating.	Bacterial count for coated samples was recorded as 3.69 and 5.51 log (CFU/mL) for *S. aureus* and *E. coli*, respectively.	[24]
Prosthetic heart valve (polystyrene-block-isobutylene-block-styrene (SIBS))	Dip coating with extracellular matrix (derived from porcine skin).	Coated samples showed improved biocompatibility and reduced protein adhesion by increasing hydrophilicity.	[25]
Abdominal Prosthetic	Polyethylene oxide coating on equine pericardium mesh.	Enzymatic degradation is more common in uncoated sample. Better mesh integrity and calcification in coated sample was observed.	[26]
Artifical Cornea	Electrospinnig of polycaprolactone and collagen.	This technique was successful in fabrication of hemispherical scaffold.	[27]
Artificial hip	Poly(2-methacryloyloxyethyl phosphorylcholine) grafted on UHMWPE	Treated samples showed reduction in wear particles.	[28]
Artificial skin	Polyimide fibers were fabricated via electrospinning, and silver nanoparticles were incorporated into the scaffold.	Silver treated samples were able to detect pressure in the range of 5 KPa to 100 KPa.	[29]
Dental implant	Dip coating of calcium carbonate on titanium.	Growth of new bone was observed after 12 days of implantation.	[30]
Artificial ligament	Polyethylene Terephthalate was dip coated with silk fibroin.	Contact angle reduced from 132° to 50°. After 12 h, DNA content of coated sample was 13 mg/scaffold, which was higher than uncoated sample (8 mg/scaffold), suggesting better biocompatability than control.	[31]
Artificial kidney	Treatment of dialysis membrane via photo-reactive zwitterionic copolymers.	Improves blood compatibility.	[32]
Dental burs	Deposition of polycrystalline diamond films via chemical vapor deposition.	Coated samples have lesser wear compared to uncoated samples.	[33]
Intramedullary nail	Coating with antibiotic and growth factors (IGF-1 and rhBMPs).	Coated samples showed controlled release of antibiotic and growth factors for development of healthy bone. Reduced risk of fracture and efficient healing were observed.	[34]
Dental crowns	Two coatings were analyzed: tribochemical silica and alumina.	Both the coated samples showed similar retention strength of 4 MPa, and control had 0.8 MPa strength.	[35]
Cochlear implant	Coating of poly(4-hydroxybutyrate) with brain derived neurotrophic factor in the presence of disuccinimidyl suberate (DSS).	Coated samples showed improved spiral ganglion cell growth	[36]
Vascular graft	Coating of gelatin/fibrinogen/polycaprolactone scaffolds prepared via electrospinning with fibronectin and collagen.	Porosity reduced to 36% for treated samples. Number of human umbilical vein endothelial cells in treated and nontreated samples was recorded as 5.71 × 10^3^ and 4.9 × 10^3^, respectively	[37]
Nikel titanium wire	Electrostatic powder technique.	Friction force was recorded as 99.65 for coated sample to, and for control it was 105.	[38]
Artificial orbital wall	Three-Dimensional printing.	Tissue volumes for pre-operation and post operation were 24 and 22.31, respectively, while for the reconstructed wall, 22.31 and 22.01 were the volumes for affected and unaffected orbit.	[39]
Titanium bone screw	Additive manufacturing using laser melting.	The mean maximal load required for fracture was 43.3 N for control and 56.6 N for treated samples.	[40]
Cartilage	Additive manufacturing of poly(ethylene oxide terephthalate/poly(butylene terephthalate) and plasma coating of acrylic acid.	Plasma coated samples showed the highest cell interaction efficiency.	[41]
Drug delivery vehicle (Nanoparticle)	Coating of gold nanoparticles with neuron-targeted exosomes.	After 30 min, 5% of treated nanoparticles were transported across the blood–brain barrier while control samples transported were less than half of treated samples.	[42]
Hernia repair	Electrospinning, plasma treatment, and direct surface modification to prepare gelatin methacryloyl and polycaprolactone methacrylate.	Treated samples showed smooth and non-defected surface. Cell viability was recorded as more than 95% for all the samples except for the polycaprolactone membrane. Treated scaffolds showed inhibitory activity against *S. aureus*, *P. aeruginosa*, and MRSA.	[43]

**Table 2:** Commonly reported infection in medical implants.

Medical Implants	Types of Materials	Infection Causing Agents	References
Orthopedic devices	Stainless steel, cobalt-based alloys, titanium, silicone, polyethylene, polypropylene, and polymethyl methacrylate; aluminium oxide and calcium phosphates.	*Acinetobacter pittii, Enterobacter cloacae, Micrococcus luteus, Staphylococcus epidermidis,* and *Staphylococcus hominis.*	[46]
Urologic devices	Polytetrafluoroethylene, rubber, polyurethane, polyamide, silicones, and polyhydroxyalkonates.	*Staphylococcus aureus* and *Staphylococcus epidermis.*	[47]
Prosthetic heart valve	Titanium, graphite, pyrolytic carbon, polyethylene, polypropylene, polyamide, and diamond-like carbon.	*Mycobacterium tuberculosis* and *hepatitis B virus.*	[48]
Bone-anchored hearing systems	Titanium, platinum, aluminium oxide, silicone, teflon, polyethylene, and polyimides.	*Staphylococcus epidermis.*	[49]
Atheoplasty devices	Cobalt-based alloys, titanium, zirconium, nickel, and ultra-high molecular weight polyethylene.	*Escherichia coli, Staphylococcus aureus,* and *Staphylococcus epidermidis.*	[50,51,52]
Bone allograft	Calcium phosphate ceramic, calcium sulphate, hydroxyapatite, bioactive glasses, and magnesium.	*Hepatitis, tuberculosis,* and *human immunodeficiency virus.*	[53]
Contact lenses and corneal implants	Silicone hydrogel and polymethylmethacrylate.	*S. epidermidis, E. coli, P. aeruginosa, S. aureus, Proteus spp., Serratia spp.,* and *Candida spp.*	[54,55,56,57,58,59,60]
Breast implants	Silicone gel within silicone rubber envelope and inflatable saline.	*S. aureus, Enterococcus spp., S. epidermidis, P. acnes, and diphtheroids.*	[61,62,63]
Dental implants	Acrylic resin, titanium and its alloys, zirconia, silver and silver nanoparticles, and ZnO.	*Veillonella spp., F. nucleatum, A. naeslundii, Streptococcus spp., C. albicans, S. sanguinis, P. gingivalis, E. timidum, E. brachy,* and *P. anerobicus.*	[58,59,64,65,66,67,68,69,70,71,72]

**Table 3:** Advantages and disadvtanges of three commonly used coating on biomaterial.

Type of Coating	Advantages	Disadvantages	References
Polymeric	Flexibility with biomaterial or biocompatible; resists corrosion and abrasion.	Low adhesive strength; permeability of body fluid across the coating.	[115,116]
Ceramic	Prevents corrosion and friction.	Comparatively heavier than organic coating; in case of cracks, corrosions occurs.	[117]
Metallic	High tensile strength; helps in osseointegration; decreases friction and wear.	Fails to show bioactivity; high elastic modulus is observed.	[118,119]

**Table 4:** Some of the biomaterials coated with antimicrobial agents via simple coating technique.

Biomaterials	Coating Materials	Method	Microorganisms Inhibited	References
Stainless steel	Ti-ZrN/Ag	Coating deposition	*S. aureus* and *S. epidermidis*	[124]
Titanium	Molybdenum disulphide	Electrostatic deposition	*E. coli*	[125]
Cotton fabrics	Zinc oxide NPs	Spin coating	*K. pneumonia*	[126]
Iron oxide NPs	Chitosan	Dip coating	*Bacillus subtilis* and *E. coli*	[127]
Nickle titanium alloy	Graphene oxide/AgNPs	Electrophoretic deposition	*S. mutans*	[128]
Ti6Al4V	Hydroxyapatite–copper	Electrophoretic deposition	*E. coli* and *S. aureus*	[129]
Carrageenan/chitosan multilayers	Nisin A	Layer by layer deposition	*S. aureus* and *MRSA*	[130]
Chitosan	N-acetyl cysteine	Spin coating	*S. aureus*	[131]

**Table 5:** Some of the significant advantages and disadvantages of plasma spray coating are mentioned in this table.

Advantages/Disadvantages		References
Advantages	Thickness of coating can be easily modulated	[148]
Disadvantages	Expensive technique; may cause crystallization of bioactive glass	[149]

**Table 6:** Some of the significant advantages and disadvantages of lithography are mentioned in this table.

Advantages/Disadvantages		References
Advantages	High accuracy	[155,156,157]
Disadvantages	It is a long process; restricted to ultraviolet; and chemically sensitive material	

**Table 7:** Some of the significant advantages and disadvantages of hydrothermal treatment are mentioned in this table.

Advantages/Disadvantages		References
Advantages	Complex shapes and geometry; cost effective; less energy consuming; mild operational temperature is needed	[161,162]
Disadvantages	Uncontrolled dispersion of coating	[163]

**Table 8:** Some of the significant advantages and disadvantages of shot peening are mentioned in this table.

Advantages/Disadvantages		References
Advantages	Improves resistance to fatigue	[166]
Disadvantages	It is not suitable for small surface area	[167]

**Table 9:** Some of the significant advantages and disadvantages of electrophoretic deposition are mentioned in this table.

Advantages/Disadvantages		References
Advantages	Complex shapes and geometry can be coated; low deposition cost	[170]
Disadvantages	Lack of uniformity	

**Table 10:** Some of the significant advantages and disadvantages of laser treatment are mentioned in this table.

Advantages/Disadvantages		References
Advantages	Requires no cooling agents and chemicals; less deformation; input of heat is low	[179]
Disadvantages	High capita cost; thickness of coating has a limit	[180]

**Table 11:** List of some of the functional agents deposited on biomaterial via electrospray deposition method.

Biomaterials	Functional Agents	Antimicrobial Effect	Results	References
**Glass**	Titanium dioxide	*S. aureus*	90% of biofilm formation was inhibited, and no viable cells were grown.	[186]
**Titanium**	Calcium silicate nanoparticles	*S. aureus* and *E. coli*	Bacterial adhesions of *S. aureus* and *E. coli* were inhibited.	[187]
**Titanium**	Vancomycin hydrochloride (VH) loaded polyvinyl alcohol-borax (PVA-B) microgels	*S. aureus*	Inhibition zone increased when increasing immersion time in normal saline.	[188]
**Chitosan/poly(ethylene glycol)/hyaluronic acid**	Zinc oxide	*S. aureus*, *S. epidermidis*, and *E. cloacea*	Inhibition zone for *S. epidermidis*, *S. aureus*, and *E. cloaceae* were recorded as 13.8, 13.0, and 10.3 mm, respectively.	[189]

**Table 12:** Some of the significant advantages and disadvantages of electrospray techniques are mentioned in this table.

Advantages/Disadvantages		References
Advantages	Motion of droplets can be controlled; prevents agglomeration and coagulation of droplets; can be used for bulk production	[190,191]
Disadvantages	May degrade macromolecules because of stress	[192]

**Table 13:** Some of the significant advantages and disadvantages of radio frequency magnetron sputtering are mentioned in this table.

Advantages/Disadvantages		References
Advantages	Dense and uniform coating is achievable; heat sensitive materials can be coated; strong adhesion of coating	[196]
Disadvantages	Costly; deposition rate is low	

**Table 14:** Some of the functional agents deposited on biomaterial via the chemical deposition method.

Biomaterials	Functional Substances	Antimicrobial Effect	Results	References
Titania Nanotube (TNT)	Silver Nanograins	*S. aureus*	TNT with 1% of silver nanograins showed 48.6% of biofilm inhibition by live/dead analysis.	[204]
Titanium	Graphene	*S. aureus* and *E. coli*	Number of colonies of *E. coli* and *S. aureus* was observed as less than 500 and 1000 CFU/mL, respectively.	[205]
Germanium	Graphene	*S. aureus* and *E. coli*	Inhibition of *E. coli* and *S. aureus* was recorded via live/dead analysis. The reports suggest that spots were visible in graphene containing samples.	[206]
Graphite	Zinc phthalocyanine	*E. coli*	97% of *E. coli* were inhibited within 15 min.	[207]
Polystyrene	1,8-cineole	*S. aureus* and *E. coli*	Fluorescence microscopy images reported that treated samples have a slightly greater number of attached *S. aureus* than *E. coli*.	[208]
Glass and latex	Antimicrobial peptide (SHAP1)	*S. aureus* and *E. coli*	More than 96% of *S. aureus and E. coli* were inhibited.	[209]
Stainless steel	Carvacrol extract	*S. aureus* and *E. coli*	More than 90% of 96% of *S. aureus and E. coli* were inhibited.	[210]

**Table 15:** Some of the significant advantages and disadvantages of chemical vapour deposition are mentioned in this table.

Advantages/Disadvantages		References
Advantages	Uniformity of film; high vacuum is not needed	[211,212,213,214,215]
Disadvantages	High capital investment is needed; chemical reactant involved can be hazardous to health; high temperature limits the use of all the substrate; highly toxic by-products; expensive instrumentation	

**Table 16:** Some of the significant advantages and disadvantages of atomic layer deposition are mentioned in this table.

Advantages/Disadvantages		References
Advantages	Requires low vacuum and low temperature; pinhole free deposition can be obtained	[217,221,222,223,224]
Disadvantages	Slow deposition rate; large amount of waste of energy and material; slow deposition rate; wastage of energy and 60% precursor	

**Table 17:** Some of the significant advantages and disadvantages of plasma immersion ion deposition are mentioned in this table.

Advantages/Disadvantages		References
Advantages	Convenient for 3D samples	[229,230]
Disadvantages	Difficulty in ion-mass separation; deposition of plasma ion onto the workpiece	

## Data Availability

Not applicable.

## References

[1] Majumdar J.D., Manna I. (2015). Laser surface engineering of titanium and its alloys for improved wear, corrosion and high-temperature oxidation resistance. Laser Surface Engineering.

[2] Hildebrand H.F. (2013). Biomaterials–a history of 7000 years. BioNanoMaterials.

[3] Ratner B.D., Zhang G. (2020). A history of biomaterials. Biomaterials Science.

[4] Crubézy E., Murail P., Girard L., Bernadou J.-P. (1998). False teeth of the Roman world. Nat. Cell Biol..

[5] Jablonská E., Kubásek J., Vojtěch D., Ruml T., Lipov J. (2021). Test conditions can significantly affect the results of in vitro cytotoxicity testing of degradable metallic biomaterials. Sci. Rep..

[6] Huang J., Best S.M. (2007). Ceramic biomaterials. Tissue Engineering Using Ceramics and Polymers.

[7] Ramakrishna S., Ramalingam M., Kumar T.S., Soboyejo W.O. (2019). Biomaterials: A Nano Approach.

[8] Prasad K., Bazaka O., Chua M., Rochford M., Fedrick L., Spoor J., Symes R., Tieppo M., Collins C., Cao A. (2017). Metallic Biomaterials: Current Challenges and Opportunities. Materials.

[9] Srivastav A. (2011). An Overview of Metallic Biomaterials for Bone Support and Replacement. Biomed. Eng. Trends Mater. Sci..

[10] Peppas N.A., Khademhosseini A. (2016). Make better, safer biomaterials. Nat. News.

[11] Park J.B., Lakes R.S. (2007). Composites as biomaterials. Biomaterials.

[12] Webber M., Appel E.A., Meijer E., Langer R. (2015). Supramolecular biomaterials. Nat. Mater..

[13] Axpe E., Orive G., Franze K., Appel E.A. (2020). Towards brain-tissue-like biomaterials. Nat. Commun..

[14] Huebsch N., Mooney D.J. (2009). Inspiration and application in the evolution of biomaterials. Nature.

[15] Gu L., Mooney D.J. (2015). Biomaterials and emerging anticancer therapeutics: Engineering the microenvironment. Nat. Rev. Cancer.

[16] Nguyen N.-Y.T., Grelling N., Wetteland C.L., Rosario R., Liu H. (2018). Antimicrobial Activities and Mechanisms of Magnesium Oxide Nanoparticles (nMgO) against Pathogenic Bacteria, Yeasts, and Biofilms. Sci. Rep..

[17] Saxena P., Pant A., Gupta S., Pant V. (2012). Release and toxicity of dental resin composite. Toxicol. Int. Former. Indian J. Toxicol..

[18] Pittet B., Montandon D., Pittet D. (2005). Infection in breast implants. Lancet Infect. Dis..

[19] Langer R., Tirrell D.A. (2004). Designing materials for biology and medicine. Nature.

[20] Hutchings I., Shipway P. (2017). Tribology: Friction and Wear of Engineering Materials.

[21] Liu Z., Liu X., Ramakrishna S. (2021). Surface engineering of biomaterials in orthopedic and dental implants: Strategies to improve osteointegration, bacteriostatic and bactericidal activities. Biotechnol. J..

[22] Zhou W., Li Y., Yan J., Xiong P., Li Q., Cheng Y., Zheng Y. (2018). Construction of Self-defensive Antibacterial and Osteogenic AgNPs/Gentamicin Coatings with Chitosan as Nanovalves for Controlled release. Sci. Rep..

[23] Kaczmarek B. (2020). Tannic acid with antiviral and antibacterial activity as a promising component of biomaterials—A mini re-view. Materials.

[24] El-Chami M.F., Mayotte J., Bonner M., Holbrook R., Stromberg K., Sohail M.R. (2020). Reduced bacterial adhesion with parylene coating: Potential implications for Micra transcatheter pacemakers. J. Cardiovasc. Electrophysiol..

[25] Wu B., Jin L., Ding K., Zhou Y., Yang L., Lei Y., Guo Y., Wang Y. (2020). Extracellular matrix coating improves the biocompatibility of polymeric heart valves. J. Mater. Chem. B.

[26] Pasculli A., Gurrado A., De Luca G.M., Mele A., Marzullo A., Mangone A., Cellamare S., Ferraro V., Maqoud F., Caggiani M.C. (2020). Bridging repair of the abdominal wall in a rat experimental model. Comparison between uncoated and polyethylene oxide-coated equine pericardium meshes. Sci. Rep..

[27] Kim J.I., Kim J.Y., Park C.H. (2018). Fabrication of transparent hemispherical 3D nanofibrous scaffolds with radially aligned patterns via a novel electrospinning method. Sci. Rep..

[28] Ishihara K. (2015). Highly lubricated polymer interfaces for advanced artificial hip joints through biomimetic design. Polym. J..

[29] Bi P., Liu X., Yang Y., Wang Z., Shi J., Liu G., Kong F., Zhu B., Xiong R. (2019). Silver-Nanoparticle-Modified Polyimide for Multiple Artificial Skin-Sensing Applications. Adv. Mater. Technol..

[30] Liu Y., Zhou Y., Jiang T., Liang Y.-D., Zhang Z., Wang Y.-N. (2017). Evaluation of the osseointegration of dental implants coated with calcium carbonate: An animal study. Int. J. Oral Sci..

[31] Jiang J., Ai C., Zhan Z., Zhang P., Wan F., Chen J., Hao W., Wang Y., Yao J., Shao Z. (2016). Enhanced fibroblast cellular liga-mentization process to polyethylene terepthalate artificial ligament by silk fibroin coating. Artif. Organs.

[32] Himmelfarb J., Ratner B. (2020). Wearable artificial kidney: Problems, progress and prospects. Nat. Rev. Nephrol..

[33] Ahmed W., Sein H., Jackson M., Rego C., Hassan I.U., Subramani K. (2018). Surface engineering of dental tools with diamond for en-hanced life and performance. Emerging Nanotechnologies in Dentistry.

[34] Berebichez-Fridman R., Montero-Olvera P., Gómez-García R., Berebichez-Fastlicht E. (2017). An intramedullary nail coated with an-tibiotic and growth factor nanoparticles: An individualized state-of-the-art treatment for chronic osteomyelitis with bone defects. Med. Hypotheses.

[35] Monteiro R.V., Dos Santos D.M., Bernardon J.K., De Souza G.M. (2020). Effect of surface treatment on the retention of zirconia crowns to tooth structure after aging. J. Esthet. Restor. Dent..

[36] Bohl A., Eickner T., Petersen S., Schmitz K.-P., Sternberg K. (2013). Specific Surface Modification of Cochlear Implant Electrode Carriers for Enhancement Of Spiral Ganglion Cell Growth. Biomed. Tech. Eng..

[37] Ardila D.C., Liou J.J., Maestas D., Slepian M.J., Badowski M., Wagner W.R., Harris D., Vande Geest J.P. (2019). Surface modification of electrospun scaffolds for endothelialization of tissue-engineered vascular grafts using human cord blood-derived endothe-lial cells. J. Clin. Med..

[38] Bandeira A.M., dos Santos M.P., Pulitini G., Elias C.N., da Costa M.F. (2011). Influence of thermal or chemical degradation on the fric-tional force of an experimental coated NiTi wire. Angle Orthod..

[39] Kang S., Kwon J., Ahn C.J., Esmaeli B., Kim G.B., Kim N., Sa H.-S. (2018). Generation of customized orbital implant templates using 3-dimensional printing for orbital wall reconstruction. Eye.

[40] Huang Y.-M., Huang C.-C., Tsai P.-I., Yang K.-Y., Huang S.-I., Shen H.-H., Lai H.-J., Huang S.-W., Chen S.-Y., Lin F.-H. (2020). Three-Dimensional Printed Porous Titanium Screw with Bioactive Surface Modification for Bone–Tendon Healing: A Rabbit Animal Model. Int. J. Mol. Sci..

[41] Cools P., Mota C., Lorenzo-Moldero I., Ghobeira R., De Geyter N., Moroni L., Morent R. (2018). Acrylic acid plasma coated 3D scaf-folds for cartilage tissue engineering applications. Sci. Rep..

[42] Khongkow M., Yata T., Boonrungsiman S., Ruktanonchai U.R., Graham D., Namdee K. (2019). Surface modification of gold nanoparti-cles with neuron-targeted exosome for enhanced blood–brain barrier penetration. Sci. Rep..

[43] Samson A., Nicole B., Vargas H.S., Sushila M., Ruiz-Esparza G.U., de Paula Mirian M.M., Webster T.J., Roberta T.C., Cruz V.B., Dan-quan W. (2021). Engineering multifunctional bactericidal nanofibers for abdominal hernia repair. Commun. Biol..

[44] Sandle T. (2019). Biocontamination Control for Pharmaceuticals and Healthcare.

[45] Koubali H., Latrache H., Zahir H., Soufiani S., El Louali M. (2020). Application of theoretical prediction to prevent the biocontamina-tion of medical materials. Proceedings of the 2020 IEEE 6th International Conference on Optimization and Applications (ICOA).

[46] Walker B., Amato C., Palyvoda O., Vangipuram S., Weaver M., Sayeed Z., Padela M.T., Yassir W.K. (2020). Prevalence of Bacterial Contamination of Casting Material in a Pediatric Population. Int. J. Pediatr..

[47] Holzapfel B.M., Reichert J.C., Schantz J.-T., Gbureck U., Rackwitz L., Nöth U., Jakob F., Rudert M., Groll J., Hutmacher D.W. (2013). How smart do biomaterials need to be? A translational science and clinical point of view. Adv. Drug Deliv. Rev..

[48] Zou S., Dodd R.Y., Stramer S.L., Strong D.M. (2004). Probability of Viremia with HBV, HCV, HIV, and HTLV among Tissue Donors in the United States. N. Engl. J. Med..

[49] Trobos M., Johansson M.L., Jonhede S., Peters H., Hoffman M., Omar O., Thomsen P., Hultcrantz M. (2018). The clinical outcome and microbiological profile of bone-anchored hearing systems (BAHS) with different abutment topographies: A prospective pi-lot study. Eur. Arch. Oto-Rhino-Laryngol..

[50] Banche G., Bracco P., Bistolfi A., Allizond V., Boffano M., Costa L., Cimino A., Cuffini A.M., del Prever E.M.B. (2011). Vitamin E blended Uhmwpe may have the potential to reduce bacterial adhesive ability. J. Orthop. Res..

[51] Banche G., Allizond V., Bracco P., Bistolfi A., Boffano M., Cimino A., Brach del Prever E.M., Cuffini A.M. (2014). Interplay be-tween surface properties of standard, vitamin E blended and oxidised ultrahigh molecular weight polyethylene used in to-tal joint replacement and adhesion of Staphylococcus aureus and *Escherichia coli*. Bone Jt. J..

[52] Banche G., Bracco P., Allizond V., Bistolfi A., Boffano M., Cimino A., Brach del Prever E.M., Cuffini A.M. (2015). Do crosslink-ing and vitamin E stabilization influence microbial adhesions on UHMWPE-based biomaterials?. Clin. Orthop. Relat. Res..

[53] Ng V.Y. (2012). Risk of Disease Transmission with Bone Allograft. Orthopedics.

[54] Cairns L.S., Marlow V.L., Bissett E., Ostrowski A., Stanley-Wall N.R. (2013). A mechanical signal transmitted by the flagellum controls signalling in B acillus subtilis. Mol. Microbiol..

[55] Belas R. (2014). Biofilms, flagella, and mechanosensing of surfaces by bacteria. Trends Microbiol..

[56] Ellison C.K., Kan J., Dillard R.S., Kysela D.T., Ducret A., Berne C., Hampton C.M., Ke Z., Wright E.R., Biais N. (2017). Obstruction of pilus retraction stimulates bacterial surface sensing. Science.

[57] An Y.H., Friedman R.J. (1998). Concise review of mechanisms of bacterial adhesion to biomaterial surfaces. J. Biomed. Mater. Res..

[58] Singh A.V., Vyas V., Patil R., Sharma V., Scopelliti P.E., Bongiorno G., Podestà A., Lenardi C., Gade W.N., Milani P. (2011). Quantitative Characterization of the Influence of the Nanoscale Morphology of Nanostructured Surfaces on Bacterial Adhesion and Biofilm Formation. PLoS ONE.

[59] Crawford R., Webb H., Truong V.K., Hasan J., Ivanova E.P. (2012). Surface topographical factors influencing bacterial attachment. Adv. Colloid Interface Sci..

[60] Zare M., Ghomi E.R., Venkatraman P.D., Ramakrishna S. (2021). Silicone-based biomaterials for biomedical applications: Antimi-crobial strategies and 3D printing technologies. J. Appl. Polym. Sci..

[61] Elbourne A., Chapman J., Gelmi A., Cozzolino D., Crawford R., Truong V.K. (2019). Bacterial-nanostructure interactions: The role of cell elasticity and adhesion forces. J. Colloid Interface Sci..

[62] Wang C., Hou J., van der Mei H.C., Busscher H.J., Ren Y. (2019). Emergent Properties in Streptococcus mutans Biofilms Are Controlled through Adhesion Force Sensing by Initial Colonizers. mBio.

[63] Viljoen A., Mignolet J., Viela F., Mathelié-Guinlet M., Dufrêne Y.F. (2020). How microbes use force to control adhesion. J. Bac-Teriol..

[64] Kokare C.R., Chakraborty S., Khopade A.N., Mahadik K.R. (2009). Biofilm: Importance and Applications. Indian J. Biotechnol..

[65] Rodrigues L., Banat I.M., Teixeira J., Oliveira R. (2007). Strategies for the prevention of microbial biofilm formation on silicone rubber voice prostheses. J. Biomed. Mater. Res. Part B Appl. Biomater..

[66] Dohnt K., Sauer M., Müller M., Atallah K., Weidemann M., Gronemeyer P., Rasch D., Tielen P., Krull R. (2011). An in vitro urinary tract catheter system to investigate biofilm development in catheter-associated urinary tract infections. J. Microbiol. Methods.

[67] Friedlander R.S., Vlamakis H., Kim P., Khan M., Kolter R., Aizenberg J. (2013). Bacterial flagella explore microscale hummocks and hollows to increase adhesion. Proc. Natl. Acad. Sci. USA.

[68] Wu S., Altenried S., Zogg A., Zuber F., Maniura-Weber K., Ren Q. (2018). Role of the Surface Nanoscale Roughness of Stainless Steel on Bacterial Adhesion and Microcolony Formation. ACS Omega.

[69] Lichter J.A., Thompson M.T., Delgadillo M., Nishikawa T., Rubner M.F., Van Vliet K.J. (2008). Substrata mechanical stiffness can regulate adhesion of viable bacteria. Biomacromolecules.

[70] Straub H., Bigger C.M., Valentin J., Abt D., Qin X.H., Eberl L., Maniura-Weber K., Ren Q. (2019). Bacterial adhesion on soft materials: Passive physicochemical interactions or active bacterial mechanosensing?. Adv. Healthc. Mater..

[71] Alam F., Balani K. (2017). Adhesion force of staphylococcus aureus on various biomaterial surfaces. J. Mech. Behav. Biomed. Mater..

[72] Gigante A., Bottegoni C., Ragone V., Banci L. (2015). Effectiveness of Vitamin-E-Doped Polyethylene in Joint Replacement: A Literature Review. J. Funct. Biomater..

[73] Allion A., Herry J.-M., Bellon-Fontaine M.-N. (2013). Material influence on biocontamination level and adhering cell physiology. MATEC Web Conf..

[74] Rosenthal V.D., Maki D.G., Graves N. (2008). The International Nosocomial Infection Control Consortium (INICC): Goals and objec-tives, description of surveillance methods, and operational activities. Am. J. Infect. Control.

[75] Persat A. (2017). Bacterial mechanotransduction. Curr. Opin. Microbiol..

[76] Han A., Tsoi J.K.H., Rodrigues F., Leprince J.G., Palin W. (2016). Bacterial adhesion mechanisms on dental implant surfaces and the influencing factors. Int. J. Adhes. Adhes..

[77] Schuldt L., Bi J., Owen G., Shen Y., Haapasalo M., Häkkinen L., Larjava H. (2021). Decontamination of rough implant surfaces colo-nized by multispecies oral biofilm by application of leukocyte-and platelet-rich fibrin. J. Periodontol..

[78] Zare M., Zare M., Butler J.A., Ramakrishna S. (2021). Nanoscience-Led Antimicrobial Surface Engineering to Prevent Infections. ACS Appl. Nano Mater..

[79] Różalska B., Sadowska B. (2018). Pet-To-Man Travelling Staphylococci.

[80] Flemming H.-C., Wingender H.-C.F.J., Szewzyk U., Steinberg P., Rice S.A., Kjelleberg S. (2016). Biofilms: An emergent form of bacterial life. Nat. Rev. Genet..

[81] Moormeier D.E., Bayles K.W. (2017). Staphylococcus aureus biofilm: A complex developmental organism. Mol. Microbiol..

[82] Rimondini L., Fini M., Giardino R. (2005). The microbial infection of biomaterials: A challenge for clinicians and researchers. A short review. J. Appl. Biomater. Biomech..

[83] Singh A., Tiwari A., Bajpai J., Bajpai A.K. (2017). Biomaterials: From Action to Application. Handbook of Antimicrobial Coatings.

[84] Zhou Y., Xiao Y., Qiu Y., Yuan H., Van Blitterswijk C.A., Zhou X., Xu X., Bao C. (2015). Adhesion and proliferation of cells and bacteria on microchip with different surfaces microstructures. Biomed. Tech. Eng..

[85] Parreira P., Magalhães A., Gonçalves I.C., Gomes J., Vidal R., Reis C.A., Leckband D.E., Martins M.C.L. (2011). Effect of surface chemistry on bacterial adhesion, viability, and morphology. J. Biomed. Mater. Res. Part A.

[86] Bose S., Robertson S.F., Bandyopadhyay A. (2018). Surface modification of biomaterials and biomedical devices using additive man-ufacturing. Acta Biomater..

[87] Arjunan A., Robinson J., Al Ani E., Heaselgrave W., Baroutaji A., Wang C. (2020). Mechanical performance of additively manufactured pure silver antibacterial bone scaffolds. J. Mech. Behav. Biomed. Mater..

[88] Wang B., Liu S., Xie Y.-Y. (2019). Role and prospects of regenerative biomaterials in the repair of spinal cord injury. Neural Regen. Res..

[89] Peppas N.A., Langer R. (1994). New Challenges in Biomaterials. Science.

[90] Biswal T., BadJena S.K., Pradhan D. (2020). Sustainable biomaterials and their applications: A short review. Mater. Today Proc..

[91] Franz S., Rammelt S., Scharnweber D., Simon J.C. (2011). Immune responses to implants—A review of the implications for the design of immunomodulatory biomaterials. Biomaterials.

[92] Wilson C.J., Clegg R.E., Leavesley D.I., Pearcy M.J. (2005). Mediation of biomaterialecell interactions by adsorbed proteins: A review. Tissue Eng..

[93] Gorbet M.B., Sefton M.V. (2004). Review: Biomaterial-associated thrombosis: Roles of coagulation factors, complement, platelets and leukocytes. Biomaterials.

[94] Schmaier A.H. (1997). Contact Activation: A Revision. Thromb. Haemost..

[95] Vogler E.A., Siedlecki C.A. (2009). Contact activation of blood-plasma coagulation. Biomaterials.

[96] Zhuo R., Siedlecki C.A., Vogler E.A. (2007). Competitive-protein adsorption in contact activation of blood factor XII. Biomaterials.

[97] Babensee J.E. (2008). Interaction of dendritic cells with biomaterials. Semin. Immunol..

[98] Sridharan R., Cameron A., Kelly D., Kearney C., O’Brien F.J. (2015). Biomaterial based modulation of macrophage polarization: A review and suggested design principles. Mater. Today.

[99] Negut I., Grumezescu V., Grumezescu A.M. (2018). Treatment Strategies for Infected Wounds. Molecules.

[100] Niroomand M.R., Arabbeiki M. (2020). Implant stability in different implantation stages: Analysis of various interface conditions. Inform. Med. Unlocked.

[101] Damm P., Bender A., Duda G., Bergmann G. (2017). In vivo measured joint friction in hip implants during walking after a short rest. PLoS ONE.

[102] Al Jabbari Y., Fournelle R., Ziebert G., Toth J., Iacopino A. (2008). Mechanical Behavior and Failure Analysis of Prosthetic Retaining Screws after Long-Term Use In Vivo. Part 2: Metallurgical and Microhardness Analysis. J. Prosthodont..

[103] Kumar R.M., Gupta P., Sharma S.K., Mittal A., Shekhar M., Kumar V., Kumar B.M., Roy P., Lahiri D. (2017). Sustained drug release from surface modified UHMWPE for acetabular cup lining in total hip implant. Mater. Sci. Eng. C.

[104] Ranuša M., Wimmer M., Fullam S., Vrbka M., Křupka I. (2021). Analysis of Friction in Total Knee Prosthesis during a Standard Gait Cycle. Lubricants.

[105] Memtsoudis S.G., Besculides M.C., Gaber L., Liu S., Della Valle A.G. (2008). Risk factors for pulmonary embolism after hip and knee arthroplasty: A population-based study. Int. Orthop..

[106] Rasool G., El Shafei Y., Stack M. (2020). Mapping Tribo-Corrosion Behaviour of TI-6AL-4V Eli in Laboratory Simulated Hip Joint Environments. Lubricants.

[107] Wos S., Koszela W., Pawlus P. (2021). Selected Methods and Applications of Anti-Friction and Anti-Wear Surface Texturing. Materials.

[108] Cui W., Qin G., Duan J., Wang H. (2017). A graded nano-TiN coating on biomedical Ti alloy: Low friction coefficient, good bonding and biocompatibility. Mater. Sci. Eng. C.

[109] Dorner-Reisel A., Schürer C., Svoboda S. (2020). Harsh Sliding Wear of a Zirconia Ball against a-C:H Coated CoCrMo Disc in Hyaluronic Gel. Lubricants.

[110] Fullam S., He J., Scholl C.S., Schmid T.M., Wimmer M.A. (2020). Competitive Binding of Bilirubin and Fatty Acid on Serum Albumin Affects Wear of UHMWPE. Lubricants.

[111] Swift K., Booker J. (2013). Manufacturing Process Selection Handbook.

[112] Rashidi H., Yang J., Shakesheff K.M. (2014). Surface engineering of synthetic polymer materials for tissue engineering and regenera-tive medicine applications. Biomater. Sci..

[113] Abela S. (2015). Physical vapour deposition on Mg alloys for biomedical applications. Surface Modification of Magnesium and Its Alloys for Biomedical Applications.

[114] Bollino F., Catauro M. (2019). Sol-Gel Technology to Prepare Advanced Coatings. Photoenergy and Thin Film Materials.

[115] Nathanael A.J., Oh T.H. (2020). Biopolymer Coatings for Biomedical Applications. Polymers.

[116] Kannan M.B. (2015). Biodegradable polymeric coatings for surface modification of magnesium-based biomaterials. Surface Modification of Magnesium and Its Alloys for Biomedical Applications.

[117] Asmatulu R. (2012). Nanocoatings for corrosion protection of aerospace alloys. Corrosion Protection and Control Using Nanomaterials.

[118] Prodana M., Stoian A.B., Burnei C., Ionita D. (2021). Innovative Coatings of Metallic Alloys Used as Bioactive Surfaces in Implantology: A Review. Coatings.

[119] Bahraminasab M., Bin Sahari B., Edwards K., Farahmand F., Arumugam M. (2012). Aseptic loosening of femoral components—Materials engineering and design considerations. Mater. Des..

[120] Wu S., Xu J., Zou L., Luo S., Yao R., Zheng B., Liang G., Wu D., Li Y. (2021). Long-lasting renewable antibacterial porous polymeric coatings enable titanium biomaterials to prevent and treat peri-implant infection. Nat. Commun..

[121] Goldschmidt G.-M., Krok-Borkowicz M., Zybała R., Pamuła E., Telle R., Conrads G., Schickle K. (2020). Biomimetic in situ precipitation of calcium phosphate containing silver nanoparticles on zirconia ceramic materials for surface functionalization in terms of antimicrobial and osteoconductive properties. Dent. Mater..

[122] Erkoc P., Ulucan-Karnak F. (2021). Nanotechnology-Based Antimicrobial and Antiviral Surface Coating Strategies. Prosthesis.

[123] Zhou J., Wang X., Zhao L. (2019). Antibacterial, angiogenic, and osteogenic activities of Ca, P, Co, F, and Sr compound doped titania coatings with different Sr content. Sci. Rep..

[124] Slate A.J., Wickens D.J., El Mohtadi M., Dempsey-Hibbert N., West G., Banks C.E., Whitehead K.A. (2018). Antimicrobial activity of Ti-ZrN/Ag coatings for use in biomaterial applications. Sci. Rep..

[125] Shin M.H., Baek S.M., Polyakov A., Semenova I.P., Valiev R.Z., Hwang W.-B., Hahn S.K., Kim H.S. (2018). Molybdenum Disulfide Surface Modification of Ultrafine-Grained Titanium for Enhanced Cellular Growth and Antibacterial Effect. Sci. Rep..

[126] Shaban M., Mohamed F., Abdallah S. (2018). Production and Characterization of Superhydrophobic and Antibacterial Coated Fabrics Utilizing ZnO Nanocatalyst. Sci. Rep..

[127] Arakha M., Pal S., Samantarrai D., Panigrahi T.K., Mallick B.C., Pramanik K., Mallick B., Jha S. (2015). Antimicrobial activity of iron ox-ide nanoparticle upon modulation of nanoparticle-bacteria interface. Sci. Rep..

[128] Pipattanachat S., Qin J., Rokaya D., Thanyasrisung P., Srimaneepong V. (2021). Biofilm inhibition and bactericidal activity of NiTi alloy coated with graphene oxide/silver nanoparticles via electrophoretic deposition. Sci. Rep..

[129] Hadidi M., Bigham A., Saebnoori E., Hassanzadeh-Tabrizi S.A., Rahmati S., Alizadeh Z.M., Nasirian V., Rafienia M. (2017). Electropho-retic-deposited hydroxyapatite-copper nanocomposite as an antibacterial coating for biomedical applications. Surf. Coat. Technol..

[130] Webber J.L., Namivandi-Zangeneh R., Drozdek S., Wilk K.A., Boyer C., Wong E.H.H., Bradshaw-Hajek B.H., Krasowska M., Beattie D.A. (2021). Incorporation and antimicrobial activity of nisin Z within carrageenan/chitosan multilayers. Sci. Rep..

[131] Costa F., Sousa D., Parreira P., Lamghari M., Gomes P., Martins M.C.L. (2017). N-acetylcysteine-functionalized coating avoids bacterial adhesion and biofilm formation. Sci. Rep..

[132] Burnett-Boothroyd S., McCarthy B. (2011). Antimicrobial treatments of textiles for hygiene and infection control applications: An industrial perspective. Textiles for Hygiene and Infection Control.

[133] Amin Yavari S., Loozen L., Paganelli F.L., Bakhshandeh S., Lietaert K., Groot J.A., Fluit A.C., Boel C.H., Alblas J., Vogely H.C. (2016). Antibacterial behavior of additively manufactured porous titanium with nanotubular surfaces releasing silver ions. ACS Appl. Mater. Interfaces.

[134] Bhadra C.M., Truong V.K., Pham V.T., Al Kobaisi M., Seniutinas G., Wang J.Y., Juodkazis S., Crawford R.J., Ivanova E.P. (2015). Antibacte-rial titanium nano-patterned arrays inspired by dragonfly wings. Sci. Rep..

[135] Jenkins J., Mantell J., Neal C., Gholinia A., Verkade P., Nobbs A.H., Su B. (2020). Antibacterial effects of nanopillar surfaces are medi-ated by cell impedance, penetration and induction of oxidative stress. Nat. Commun..

[136] Elbourne A., Coyle V.E., Truong V.K., Sabri Y.M., Kandjani A.E., Bhargava S.K., Ivanova E.P., Crawford R.J. (2019). Multi-directional elec-trodeposited gold nanospikes for antibacterial surface applications. Nanoscale Adv..

[137] Li X. (2015). Bactericidal mechanism of nanopatterned surfaces. Phys. Chem. Chem. Phys..

[138] Francolini I., Vuotto C., Piozzi A., Donelli G. (2017). Antifouling and antimicrobial biomaterials: An overview. APMIS.

[139] Rainsford K.D., Powanda M.C., Whitehouse M.W. (2015). Preface. Novel Natural Products: Therapeutic Effects in Pain Arthritis and Gastro-intestinal Diseases. Prog. Drug Res. Fortschr. Arzneimittelforschung. Prog. Rech. Pharm..

[140] Denyer S.P. (1995). Mechanisms of action of antibacterial biocides. Int. Biodeterior. Biodegrad..

[141] Stepnova E.A., Tikhonov V.E., Babushkina T.A., Klimova T.P., Vorontsov E.V., Babak V.G., Lopatin S.A., Yamskov I.A. (2007). New approach to the quaternization of chitosan and its amphiphilic derivatives. Eur. Polym. J..

[142] Barros J., Dias A., Rodrigues M.A., Pina-Vaz C., Lopes M.A., Pina-Vaz I. (2015). Antibiofilm and Antimicrobial Activity of Polyethyl-enimine: An Interesting Compound for Endodontic Treatment. J. Contemp. Dent. Pract..

[143] Leyland N.S., Podporska-Carroll J., Browne J., Hinder S.J., Quilty B., Pillai S.C. (2016). Highly Efficient F, Cu doped TiO2 anti-bacterial visible light active photocatalytic coatings to combat hospital-acquired infections. Sci. Rep..

[144] Makhlouf A. (2011). Current and advanced coating technologies for industrial applications. Nanocoatings and Ultra-Thin Films.

[145] Fauchais P., Vardelle A., Dussoubs B. (2001). Quo vadis thermal spraying?. J. Therm. Spray Technol..

[146] Pillai R.S., Frasnelli M., Sglavo V.M. (2018). HA/β-TCP plasma sprayed coatings on Ti substrate for biomedical applications. Ceram. Int..

[147] Liang R., Xu Y., Zhao M., Han G., Li J., Wu W., Dong M., Yang J., Liu Y. (2019). Properties of silver contained coatings on CoCr alloys prepared by vacuum plasma spraying. Mater. Sci. Eng. C.

[148] Daroonparvar M., Yajid M.A., Yusof N.M., Bakhsheshi-Rad H.R., Hamzah E., Mardanikivi T. (2015). Deposition of duplex MAO layer/nanostructured titanium dioxide composite coatings on Mg–1% Ca alloy using a combined technique of air plasma spraying and micro arc oxidation. J. Alloys Compd..

[149] Sergi R., Bellucci D., Cannillo V. (2020). A Comprehensive Review of Bioactive Glass Coatings: State of the Art, Challenges and Future Perspectives. Coatings.

[150] Gowan A., Meggs P.B., Ashwin C. (1984). A History of Graphic Design. Des. Issues.

[151] Subramani K., Ahmed W. (2012). Fabrication of PEG Hydrogel Micropatterns by Soft-Photolithography and PEG Hydrogel as Guided Bone Regeneration Membrane in Dental Implantology. Emerging Nanotechnologies in Dentistry.

[152] Tran K.T., Nguyen T.D. (2017). Lithography-based methods to manufacture biomaterials at small scales. J. Sci. Adv. Mater. Devices.

[153] Qin D., Xia Y., Whitesides G.M. (2010). Soft lithography for micro- and nanoscale patterning. Nat. Protoc..

[154] Arango-Santander S., Pelaez-Vargas A., Freitas S.C., García C. (2018). A novel approach to create an antibacterial surface using tita-nium dioxide and a combination of dip-pen nanolithography and soft lithography. Sci. Rep..

[155] Bishop E.S., Mostafa S., Pakvasa M., Luu H.H., Lee M.J., Wolf J.M., Ameer G.A., He T.-C., Reid R.R. (2017). 3-D bioprinting technologies in tissue engineering and regenerative medicine: Current and future trends. Genes Dis..

[156] Guvendiren M., Molde J., Soares R.M., Kohn J. (2016). Designing Biomaterials for 3D Printing. ACS Biomater. Sci. Eng..

[157] Li J., Chen M., Fan X., Zhou H. (2016). Recent advances in bioprinting techniques: Approaches, applications and future prospects. J. Transl. Med..

[158] Xu Q.-T., Li J.-C., Xue H.-G., Guo S.-P. (2018). Binary iron sulfides as anode materials for rechargeable batteries: Crystal structures, syntheses, and electrochemical performance. J. Power Source.

[159] Cao Y., Su B., Chinnaraj S., Jana S., Bowen L., Charlton S., Duan P., Jakubovics N., Chen J. (2018). Nanostructured titanium surfaces exhibit recalcitrance towards Staphylococcus epidermidis biofilm formation. Sci. Rep..

[160] Wang J., Li J., Guo G., Wang Q., Tang J., Zhao Y., Qin H., Wahafu T., Shen H., Liu X. (2016). Silver-nanoparticles-modified bio-material surface resistant to staphylococcus: New insight into the antimicrobial action of silver. Sci. Rep..

[161] Zuldesmi M., Waki A., Kuroda K., Okido M. (2013). High Osteoconductive Surface of Pure Titanium by Hydrothermal Treatment. J. Biomater. Nanobiotechnol..

[162] Gao Y., Remón J., Matharu A.S. (2021). Microwave-assisted hydrothermal treatments for biomass valorisation: A critical review. Green Chem..

[163] Zhang T., Xiao X. (2020). Hydrothermal Synthesis of Hydroxyapatite Assisted by Gemini Cationic Surfactant. J. Nanomater..

[164] Kirk D. (1999). Shot peening. Aircr. Eng. Aerosp. Technol..

[165] Nino-Barrera J., Sanchez-Aleman J., Acosta-Humanez M., Gamboa-Martinez L., Cortes-Rodriguez C. (2021). Shot peening increases resistance to cyclic fatigue fracture of endodontic files. Sci. Rep..

[166] Verdian M. (2017). 3.13 Finishing and Post-Treatment of Thermal Spray Coatings.

[167] Irizalp S.G., Saklakoglu N. (2017). 1.14 Laser Peening of Metallic Materials.

[168] Boccaccini A.R., Keim S., Ma R., Li Y., Zhitomirsky I. (2010). Electrophoretic deposition of biomaterials. J. R. Soc. Interface.

[169] Chen C.Y., Kim D.M., Lee C., Da Silva J., Nagai S., Nojiri T., Nagai M. (2020). Biological efficacy of perpendicular type-I collagen pro-truded from TiO 2-nanotubes. Int. J. Oral Sci..

[170] Bensebaa F. (2013). Nanoparticle fundamentals. Interface Science and Technology.

[171] Char D.S., Shah N.H., Magnus D. (2018). Implementing Machine Learning in Health Care—Addressing Ethical Challenges. N. Engl. J. Med..

[172] Ebrahiminezhad A., Raee M.J., Manafi Z., Jahromi A.S., Ghasemi Y. (2016). Ancient and Novel Forms of Silver in Medicine and Biomedicine. J. Adv. Med Sci. Appl. Technol..

[173] Baranwal A., Srivastava A., Kumar P., Bajpai V.K., Maurya P.K., Chandra P. (2018). Prospects of Nanostructure Materials and Their Composites as Antimicrobial Agents. Front. Microbiol..

[174] Nene A.G., Galluzzi M., Luo H., Somani P., Ramakrishna S., Yu X.F. (2021). Synthetic preparations and atomic scale engineering of sil-ver nanoparticles for biomedical applications. Nanoscale.

[175] Abi-Gerges A., Fischmeister R. (2013). Ion Channels: New Tools to Track Cyclic Nucleotide Changes in Living Cells. Encyclopedia of Biophysics.

[176] Bandyopadhyay A., Balla V.K., Roy M., Bose S. (2011). Laser surface modification of metallic biomaterials. JOM.

[177] Luo X., Yao S., Zhang H., Cai M., Liu W., Pan R., Chen C., Wang X., Wang L., Zhong M. (2019). Biocompatible nano-ripples structured surfaces induced by femtosecond laser to rebel bacterial colonization and biofilm formation. Opt. Laser Technol..

[178] Lutey A.H.A., Gemini L., Romoli L., Lazzini G., Fuso F., Faucon M., Kling R. (2018). Towards Laser-Textured Antibacterial Surfaces. Sci. Rep..

[179] Babić M., Balic J., Milfelner M., Belič I., Kokol P., Zorman M., Panjan P. (2013). Robot laser hardening and the problem of overlapping laser beam. Adv. Prod. Eng. Manag..

[180] Narayan R., Goering P. (2011). Laser micro- and nanofabrication of biomaterials. MRS Bull..

[181] Babič M. (2021). Modeling surface roughness of point robot laser hardening, with emphasis on the surface. Politehnika.

[182] Avilez H.R., Casadiego D.C., Avila A.V., Perez O.P., Almodovar J. (2017). Production of chitosan coatings on metal and ceramic bio-materials. Chitosan Based Biomaterials.

[183] Lei L., Gamboa A.R., Kuznetsova C., Littlecreek S., Wang J., Zou Q., Zahn J.D., Singer J.P. (2020). Self-limiting electrospray deposition on polymer templates. Sci. Rep..

[184] Boda S.K., Li X., Xie J. (2018). Electrospraying an enabling technology for pharmaceutical and biomedical applications: A review. J. Aerosol Sci..

[185] Im S.-Y., Kim K.-M., Kwon J.-S. (2020). Antibacterial and Osteogenic Activity of Titania Nanotubes Modified with Electrospray-Deposited Tetracycline Nanoparticles. Nanomaterials.

[186] Jalvo B., Faraldos M., Bahamonde A., Rosal R. (2018). Antibacterial surfaces prepared by electrospray coating of photocatalytic na-noparticles. Chem. Eng. J..

[187] Buga C., Chen C.-C., Hunyadi M., Csík A., Hegedűs C., Ding S.-J. (2021). Electrosprayed calcium silicate nanoparticle-coated titanium implant with improved antibacterial activity and osteogenesis. Colloids Surf. B Biointerfaces.

[188] Lv J., Li X., Yin H., Wang L., Pei Y., Lv X. (2017). Controlled release of vancomycin hydrochloride from a composite structure of pol-ymeric films and porous fibers on implants. Chem. Eng. J..

[189] Mutlu E.C., Yıldırım A.B., Yıldırım M., Ficai A., Ficai D., Oktar F.N., Ţîţu M., Çetinkaya A., Demir A. (2020). Improvement of antibacteri-al and biocompatibility properties of electrospray biopolymer films by ZnO and MCM-41. Polym. Bull..

[190] Jaworek A. (2016). Electrohydrodynamic microencapsulation technology. Encapsulations.

[191] Jaworek A. (2007). Micro- and nanoparticle production by electrospraying. Powder Technol..

[192] Sridhar R., Ramakrishna S. (2013). Electrosprayed nanoparticles for drug delivery and pharmaceutical applications. Biomatter.

[193] Surmenev R., Vladescu A., Surmeneva M., Ivanova M.B.A., Grubova I., Cotrut C.M. (2017). Radio Frequency Magnetron Sputter Deposition as a Tool for Surface Modification of Medical Implants. Modern Technologies for Creating the Thin-Film Systems and Coatings.

[194] Hassan M.M. (2018). Antimicrobial Coatings for Textiles. Handbook of Antimicrobial Coatings.

[195] Hao J., Li Y., Liao R., Liu G., Liao Q., Tang C. (2017). Fabrication of Al2O3 Nano-Structure Functional Film on a Cellulose Insulation Polymer Surface and Its Space Charge Suppression Effect. Polymers.

[196] Piedade A., Pinho A.C., Branco R., Morais P. (2020). Evaluation of antimicrobial activity of ZnO based nanocomposites for the coating of non-critical equipment in medical-care facilities. Appl. Surf. Sci..

[197] Sun L., Yuan G., Gao L., Yang J., Chhowalla M., Gharahcheshmeh M.H., Gleason K.K., Choi Y.S., Hong B.H., Liu Z. (2021). Chemical va-pour deposition. Nat. Rev. Methods Primers.

[198] Pierson H.O. (1999). Handbook of Chemical Vapor Deposition: Principles, Technology and Applications.

[199] Sanders S., Stümmler D., Pfeiffer P., Ackermann N., Simkus G., Heuken M., Baumann P.K., Vescan A., Kalisch H. (2019). Chemical Vapor Deposition of Organic-Inorganic Bismuth-Based Perovskite Films for Solar Cell Application. Sci. Rep..

[200] Alotaibi A.M., Sathasivam S., Nair S.P., Parkin I.P. (2015). Antibacterial properties of Cu–ZrO2thin films prepared via aerosol assisted chemical vapour deposition. J. Mater. Chem. B.

[201] Su C., Hu Y., Song Q., Ye Y., Gao L., Li P., Ye T. (2020). Initiated chemical vapor deposition of graded polymer coatings enabling anti-bacterial, antifouling, and biocompatible surfaces. ACS Appl. Mater. Interfaces.

[202] Fang C., Cao Y., Wu D., Li A. (2018). Thermal atomic layer etching: Mechanism, materials and prospects. Prog. Nat. Sci. Mater. Int..

[203] Antoun G., Tillocher T., Lefaucheux P., Faguet J., Maekawa K., Dussart R. (2021). Mechanism understanding in cryo atomic layer etching of SiO2 based upon C4F8 physisorption. Sci. Rep..

[204] Piszczek P., Lewandowska Ż., Radtke A., Jędrzejewski T., Kozak W., Sadowska B., Szubka M., Talik E., Fiori F. (2017). Biocompatibility of Titania Nanotube Coatings Enriched with Silver Nanograins by Chemical Vapor Deposition. Nanomaterials.

[205] Gu M., Lv L., Du F., Niu T., Chen T., Xia D., Wang S., Zhao X., Liu J., Liu Y. (2018). Effects of thermal treatment on the adhesion strength and osteoinductive activity of single-layer graphene sheets on titanium substrates. Sci. Rep..

[206] Geng H., Dai J., Li J., Di Z., Liu X. (2016). Antibacterial ability and hemocompatibility of graphene functionalized germanium. Sci. Rep..

[207] Arellano D.L.G., Kolewe K.W., Champagne V.K., Kurtz I.S., Burnett E.K., Zakashansky J., Arisoy F.D., Briseno A.L., Schiffman J.D. (2018). Gecko-Inspired Biocidal Organic Nanocrystals Initiated from a Pencil-Drawn Graphite Template. Sci. Rep..

[208] Mann M.N., Fisher E.R. (2017). Investigation of Antibacterial 1,8-Cineole-Derived Thin Films Formed via Plasma-Enhanced Chemical Vapor Deposition. ACS Appl. Mater. Interfaces.

[209] Jeong G.M., Seong H., Im S.G., Sung B.H., Kim S.C., Jeong K.J. (2018). Coating of an antimicrobial peptide on solid substrate via initiated chemical vapor deposition. J. Ind. Eng. Chem..

[210] Getnet T.G., da Silva G.F., S Duarte I., Kayama M.E., Rangel E.C., Cruz N.C. (2020). Atmospheric Pressure Plasma Chemical Vapor Depo-sition of Carvacrol Thin Films on Stainless Steel to Reduce the Formation of *E. Coli* and *S. Aureus* Biofilms. Materials.

[211] Raiford J.A., Oyakhire S.T., Bent S.F. (2020). Applications of atomic layer deposition and chemical vapor deposition for perovskite solar cells. Energy Environ. Sci..

[212] Rockett A. (2008). The Materials Science of Semiconductors. Mater. Sci. Semicond..

[213] Yan X.T., Xu Y. (2010). Chemical Vapour Deposition: An Integrated Engineering Design for Advanced Materials.

[214] Malekimoghadam R., Rafiee R. (2018). Carbon nanotubes processing. Carbon Nanotube-Reinforced Polymers.

[215] Karfa P., Majhi K.C., Madhuri R. (2020). Synthesis of two-dimensional nanomaterials. Two-Dimensional Nanostructures for Biomedical Technology.

[216] Lee J.H., Kim U.J., Han C.H., Rha S.K., Lee W.J., Park C.O. (2004). Investigation of silicon oxide thin films prepared by atomic layer dep-osition using SiH2Cl2 and O3 as the precursors. Jpn. J. Appl. Phys..

[217] Johnson R.W., Hultqvist A., Bent S.F. (2014). A brief review of atomic layer deposition: From fundamentals to applications. Mater. Today.

[218] Hu L., Qi W., Li Y. (2018). Coating strategies for atomic layer deposition. Nanotechnol. Rev..

[219] Narayan R.J., Adiga S.P., Pellin M.J., A Curtiss L., Stafslien S., Chisholm B., A Monteiro-Riviere N., Brigmon R.L., Elam J.W. (2010). Atomic layer deposition of nanoporous biomaterials. Mater. Today.

[220] Liu L., Bhatia R., Webster T.J. (2017). Atomic layer deposition of nano-TiO2 thin films with enhanced biocompatibility and antimi-crobial activity for orthopedic implants. Int. J. Nanomed..

[221] Sutherland B.R., Hoogland S., Adachi M.M., Kanjanaboos P., Wong C.T.O., McDowell J.J., Xu J., Voznyy O., Ning Z., Houtepen A.J. (2014). Perovskite Thin Films via Atomic Layer Deposition. Adv. Mater..

[222] Leskelä M., Ritala M. (2003). Atomic Layer Deposition Chemistry: Recent Developments and Future Challenges. Angew. Chem. Int. Ed..

[223] Muñoz-Rojas D., MacManus-Driscoll J. (2014). Spatial atmospheric atomic layer deposition: A new laboratory and industrial tool for low-cost photovoltaics. Mater. Horiz..

[224] Oviroh P.O., Akbarzadeh R., Pan D., Coetzee R.A.M., Jen T.-C. (2019). New development of atomic layer deposition: Processes, methods and applications. Sci. Technol. Adv. Mater..

[225] Anders A. (1997). Metal plasma immersion ion implantation and deposition: A review. Surf. Coat. Technol..

[226] Mantese J.V., Brown I.G., Cheung N.W., Collins G.A. (1996). Plasma-immersion ion implantation. Mrs Bull..

[227] Harrasser N., Jüssen S., Obermeir A., Kmeth R., Stritzker B., Gollwitzer H., Burgkart R. (2016). Antibacterial potency of different depo-sition methods of silver and copper containing diamond-like carbon coated polyethylene. Biomater. Res..

[228] Shiau D.K., Yang C.H., Sun Y.S., Wu M.F., Pan H., Huang H.H. (2019). Enhancing the blood response and antibacterial adhesion of tita-nium surface through oxygen plasma immersion ion implantation treatment. Surf. Coat. Technol..

[229] Pelletier J., Anders A. (2005). Plasma-based ion implantation and deposition: A review of physics, technology, and applications. IEEE Trans. Plasma Sci..

[230] Wood B.P. (2000). Fundamentals of Plasma Immersion Ion Implantation and Deposition. https://escholarship.org/uc/item/4pz675zk.

[231] Liu C.-F., Lee T.-H., Liu J.-F., Hou W.-T., Li S.-J., Hao Y.-L., Pan H., Huang H.-H. (2018). A unique hybrid-structured surface produced by rapid electrochemical anodization enhances bio-corrosion resistance and bone cell responses of β-type Ti-24Nb-4Zr-8Sn alloy. Sci. Rep..

[232] Lee H.-S., Singh J.K., Ismail M.A., Bhattacharya C., Seikh A.H., Alharthi N., Hussain R.R. (2019). Corrosion mechanism and kinetics of Al-Zn coating deposited by arc thermal spraying process in saline solution at prolong exposure periods. Sci. Rep..

[233] Jones A., Mistry K., Kao M., Shahin A., Yavuz M., Musselman K.P. (2020). In-situ spatial and temporal electrical characterization of ZnO thin films deposited by atmospheric pressure chemical vapour deposition on flexible polymer substrates. Sci. Rep..

[234] Li W., Su P., Li Z., Xu Z., Wang F., Ou H., Zhang J., Zhang G., Zeng E. (2017). Ultrathin metal–organic framework membrane production by gel–vapour deposition. Nat. Commun..

[235] Pessoa R., dos Santos V., Cardoso S., Doria A., Figueira F., Rodrigues B., Testoni G., Fraga M., Marciano F., Lobo A.O. (2017). TiO2 coatings via atomic layer deposition on polyurethane and polydimethylsiloxane substrates: Properties and effects on C. albicans growth and inactivation process. Appl. Surf. Sci..

[236] Beigi M.H., Safaie N., Nasr-Esfahani M.H., Kiani A. (2019). 3D titania nanofiber-like webs induced by plasma ionization: A new direc-tion for bioreactivity and osteoinductivity enhancement of biomaterials. Sci. Rep..

[237] Yu K., Mei Y., Hadjesfandiari N., Kizhakkedathu J.N. (2014). Engineering biomaterials surfaces to modulate the host response. Colloids Surf. B Biointerfaces.

[238] Fundeanu I., van der Mei H.C., Schouten A.J., Busscher H.J. (2010). Microbial adhesion to surface-grafted polyacrylamide brushes after long-term exposure to PBS and reconstituted freeze-dried saliva. J. Biomed. Mater. Res. Part A.

[239] Yang W.J., Cai T., Neoh K.-G., Kang E.-T., Teo S.L.-M., Rittschof D. (2013). Barnacle Cement as Surface Anchor for “Clicking” of Antifouling and Antimicrobial Polymer Brushes on Stainless Steel. Biomacromolecules.

[240] Kowalski P.S., Bhattacharya C., Afewerki S., Langer R.S. (2018). Smart Biomaterials: Recent Advances and Future Directions. ACS Biomater. Sci. Eng..

[241] Montoya C., Du Y., Gianforcaro A.L., Orrego S., Yang M., Lelkes P.I. (2021). On the road to smart biomaterials for bone research: Defi-nitions, concepts, advances, and outlook. Bone Res..

[242] Hager M.D., Greil P., Leyens C., van der Zwaag S., Schubert U.S. (2010). Self-healing materials. Adv. Mater..

[243] Diba M., Spaans S., Ning K., Ippel B.D., Yang F., Loomans B., Dankers P.Y.W., Leeuwenburgh S.C.G. (2018). Self-Healing Biomaterials: From Molecular Concepts to Clinical Applications. Adv. Mater. Interfaces.

[244] Chen Y., Kushner A.M., Williams G.A., Guan Z. (2012). Multiphase design of autonomic self-healing thermoplastic elastomers. Nat. Chem..

[245] White S.R., Sottos N.R., Geubelle P.H., Moore J.S., Kessler M.R., Sriram S.R., Brown E.N., Viswanathan S. (2001). Autonomic healing of polymer composites. Nature.

